# Rod nuclear architecture determines contrast transmission of the retina and behavioral sensitivity in mice

**DOI:** 10.7554/eLife.49542

**Published:** 2019-12-11

**Authors:** Kaushikaram Subramanian, Martin Weigert, Oliver Borsch, Heike Petzold, Alfonso Garcia-Ulloa, Eugene W Myers, Marius Ader, Irina Solovei, Moritz Kreysing

**Affiliations:** 1 Max Planck Institute of Molecular Cell Biology and GeneticsDresdenGermany; 2 Center for Systems Biology DresdenDresdenGermany; 3 Cluster of Excellence, Physics of Life, Technische Universität DresdenDresdenGermany; 4 Center for Regenerative Therapies DresdenTechnische Universität DresdenDresdenGermany; 5 Department of Computer ScienceTechnische Universität DresdenDresdenGermany; 6 Biozentrum, Ludwig Maximilians UniversitätMünchenGermany; Johns Hopkins University School of MedicineUnited States; Emory UniversityUnited States

**Keywords:** biophysics, vision, retina, optics, Chromatin organisation, visual ecology, Mouse

## Abstract

Rod photoreceptors of nocturnal mammals display a striking inversion of nuclear architecture, which has been proposed as an evolutionary adaptation to dark environments. However, the nature of visual benefits and the underlying mechanisms remains unclear. It is widely assumed that improvements in nocturnal vision would depend on maximization of photon capture at the expense of image detail. Here, we show that retinal optical quality improves 2-fold during terminal development, and that this enhancement is caused by nuclear inversion. We further demonstrate that improved retinal contrast transmission, rather than photon-budget or resolution, enhances scotopic contrast sensitivity by 18–27%, and improves motion detection capabilities up to 10-fold in dim environments. Our findings therefore add functional significance to a prominent exception of nuclear organization and establish retinal contrast transmission as a decisive determinant of mammalian visual perception.

## Introduction

**Figure 1.**
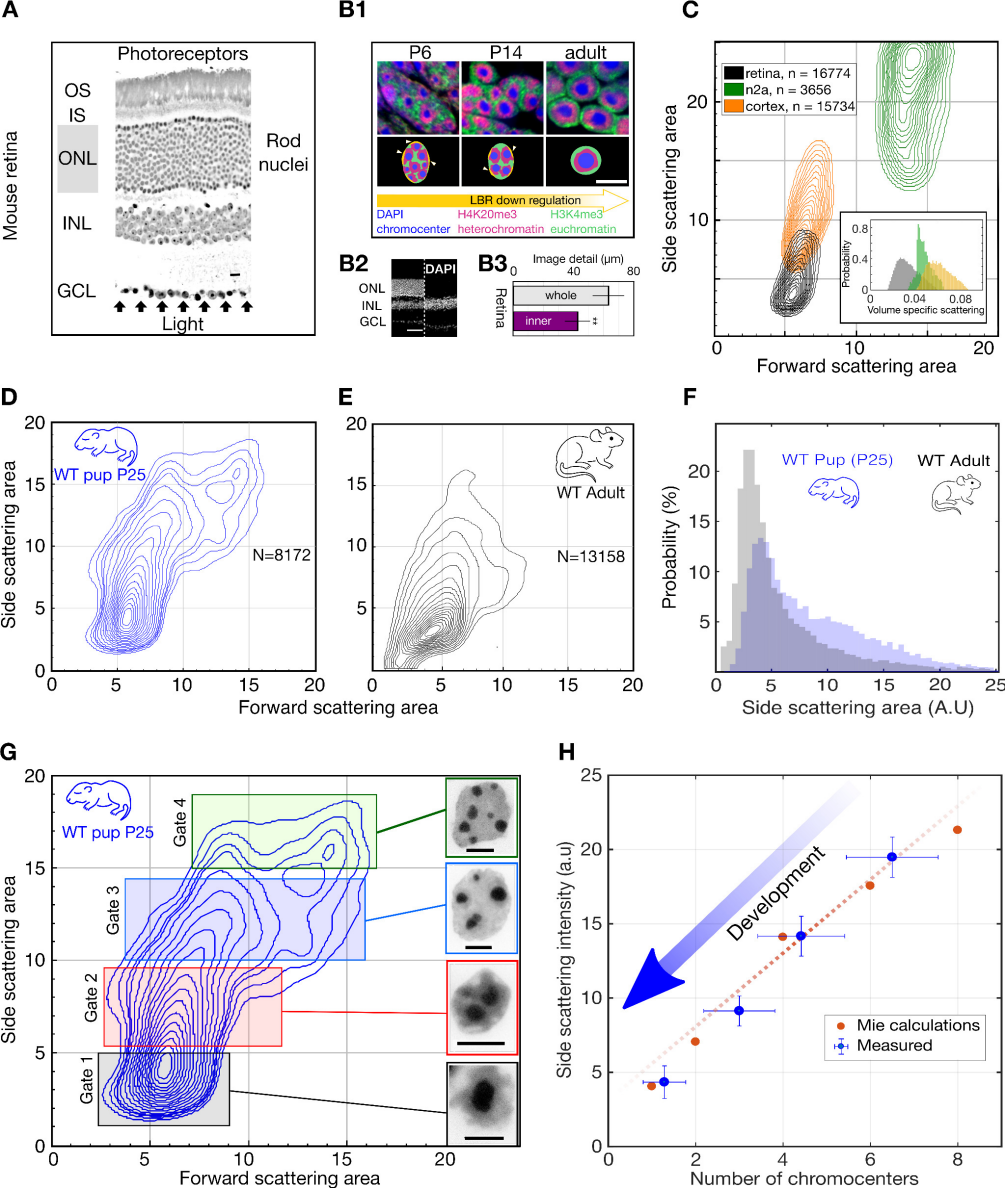
Light scattering by retinal nuclei reduces with chromocenter number during development. (**A**) Longitudinal section showing the path of light through the mouse retina, including the rod nuclei dominated outer nuclear layer (ONL). Ganglion cell layer (GCL), inner nuclear layer (INL) and outer nuclear layer (ONL) and the inner and outer segments (IS and OS). (**B1**) (top) Downregulation of the lamina tether LBR (yellow) enables fusion of mobilized chromocenters and thereby an architectural inversion of mouse rod nuclei. (bottom) FISH images of rod nuclei stained with DAPI (blue) showing the dense chromocenters, LINE rich heterochromatin (H4K20me3, magenta) and SINE rich euchromatin (H3K4me3, green) (**B2**) DAPI section of WT mouse retina in comparison to a Rd1/Cpfl1-KO mouse retina showing the presence of only the inner retina. (**B3**) Quantification of image transmission shows that the inner retina alone (Rd1/Cpfl1-KO, N = 5) *transmits approximately 50% more image* detail than the full retina (N = 11), suggesting significant image degradation in the thick outer nuclear layer. (**C**) FACS scattering profiles comparing retinal neurons, cortical neurons and N2a neuroblastoma cells showing lower light scattering properties of retina neurons. (Inset) Volume-specific light scattering is significantly reduced in the retinal cell nuclei. (**D, E**) FACS scatter plot for isolated retinal nuclei from WT developmental stage week three pup (P25) and adult mice demonstrating stronger large angle scattering by the P25 nuclei. (**F**) Histogram of side scattering in adult and P25 retina depicting a higher side scattering for the developing retinal nuclei. (**G**) Sorting of developmentally maturing nuclei according to different side scattering signal. Insets show representative examples of Hoechst stained nuclei in the corresponding sort fractions. The rectangles represent sorting gates for microscopy analysis. (**H**) Quantification of reduced scattering with chromocenter number is sufficiently explained by a wave optical model of light scattering n = 38 nuclei. (Error bars in (**H**) show s.d.) Scale bars (**A**) - 10 μm. (B1), G - 5 μm, (B2) – 50 µm.

The structure of the vertebrate retina requires light to pass through multiple cell layers prior to reaching the light-sensitive outer segments of the photoreceptors (Dowling, 1987). In nocturnal mammals, the increased density of rod photoreceptor cells demands a thicker (Němec et al., 2007; Peichl, 2005) rod nuclei-containing outer nuclear layer (ONL). For mice, where rods account for around 80% of all retinal cells (Hughes et al., 2017), this layer of photoreceptor nuclei is 55 ± 5 µm thick, thus creating an apparent paradox by acting as a more pronounced barrier for projected images prior to their detection (Figure 1A). Interestingly, rod nuclei are inverted in nocturnal mammals (Błaszczak et al., 2014; Falk et al., 2019; Kreysing et al., 2010; Solovei et al., 2009; Solovei et al., 2013) such that heterochromatin is detached from the nuclear envelope and found in the nuclear center, whereas euchromatin that has lower mass density (Imai et al., 2017) is re-located to the nuclear periphery. Given that this nuclear inversion is exclusive to nocturnal mammals and correlates with the light-focusing capabilities of isolated nuclei, it was proposed as an evolutionary adaptation to life under low-light conditions (Błaszczak et al., 2014; Kreysing et al., 2010; Solovei et al., 2009). However, the nature of any visual improvements that could arise from nuclear inversion remains unclear.

**Figure 1—figure supplement 1.**
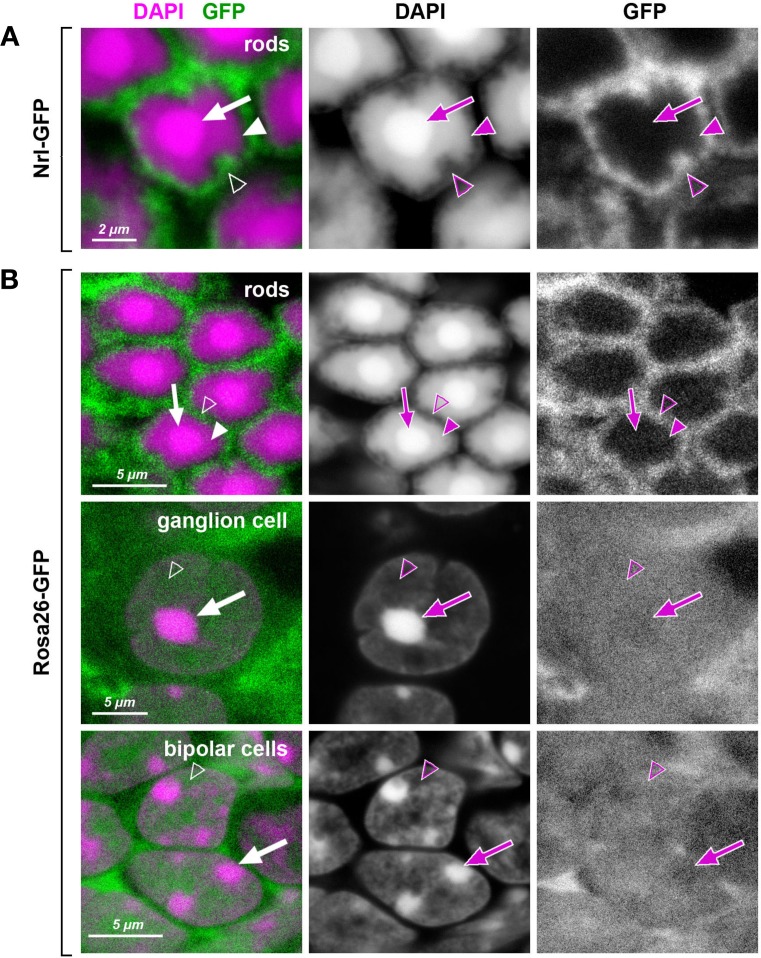
Heterochromatin in mouse rod nuclei exhibits unusual dense packing. Retinal cells of transgenic mice expressing GFP (green) under rod-specific Nrl promoter (**A**; Akimoto et al., 2006) and under control of the ROSA26 promoter (**B**; Ivanova et al., 2005). In inverted rod nuclei, the chromatin of the central chromocenter (arrows) and the surrounding shell of LINE-rich heterochromatin (arrowheads) is packed so densely that free molecules of GFP do not penetrate into these nuclear regions. In contrast, loosely packed euchromatin in the peripheral nuclear shell (empty arrowheads) allow GFP penetration. In conventional nuclei, exemplified by ganglion and bipolar cells, the entire nucleoplasm, regardless to chromatin nature, is penetrable for GFP with chromocenters showing slightly less permeability (arrows). Nuclei are counterstained with DAPI (magenta). Single confocal sections.

**Figure 1—figure supplement 2.**
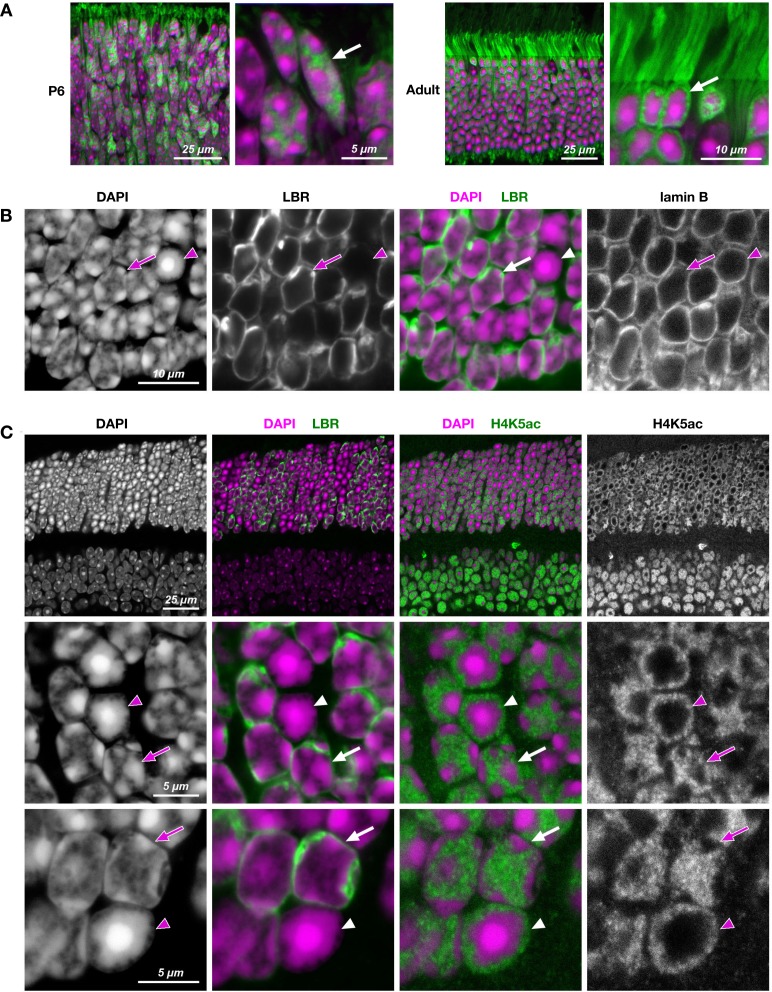
Reorganization of rod nuclear architecture in the course of postnatal retinal development (**A**) and in transgenic rods expressing LBR (**B, C**). (**A**) Difference in nuclear architecture of terminally differentiated rods (adult,) and photoreceptor progenitors (P6) is highlighted by GFP (green) expressed under Nrl promoter and freely distributed through nucleoplasm and cytoplasm. During first 4–6 weeks of postnatal development, conventional nuclear architecture of rod progenitors (arrow), characterized by multiple chromocenters adjacent to the nuclear periphery, is gradually rearranged into inverted one of fully mature rods (arrow) with a single central chromocenter surrounded by LINE-rich heterochromatin. (**B, C**) Rod nuclei ectopically expressing LBR (green) in adult TG-LBR retina have conventional nuclear organization with chromocenters adjacent to the nuclear lamina (**B**) and euchromatin occupying the nuclear interior (**C**). Nuclear lamina is stained with antibodies to lamin B (**B**) and euchromatin is highlighted by H4K5ac staining (**C**). Note that only proportion of rods in TG-LBR retina express LBR and thus maintain conventional nuclei (arrows). Nuclei of rods not expressing LBR are lacking peripheral tethers of heterochromatin and ultimately undergo inversion (arrowheads). Nuclei are counterstained with DAPI (magenta). Single confocal sections.

It is widely assumed that high-sensitivity vision depends on optimized photon capture (Schmucker and Schaeffel, 2004; Warrant and Locket, 2004) and often comes at the expense of image detail (Cronin et al., 2014; Warrant, 1999). Here, we show that nuclear inversion affects a different metric of vision, namely contrast sensitivity under low-light conditions. In particular, we experimentally show that nuclear inversion improves retinal contrast transmission, rather than photon capture or resolution. Advanced optical modelling and large-angle scattering measurements indicate that this enhanced contrast transfer emerges from previously coarse-grained (Błaszczak et al., 2014; Kreysing et al., 2010; Solovei et al., 2009) changes in nuclear granularity, namely a developmental reduction of chromocenter number (Figure 1B1). Moreover, genetic interventions to change chromocenter number in adult mice reduces contrast transmission through the retina, and compromise nocturnal contrast sensitivity accordingly. Our study therefore adds functional significance to nuclear inversion by establishing retinal contrast transmission as a decisive determinant of mammalian vision.

## Results

### Volume-specific light scattering from chromocenters

To test how the presence of densely packed rod nuclei in the light path affects the propagation of light through the retina, we compared transmission of micro-projected stripe images through freshly excised retinae of wild type (WT) (Figure 1A, Figure 1B2 - left image) and Rd1/Cpfl1- KO mice (Chang et al., 2002), which lack all photoreceptors including the ONL (Figure 1B2 - right image). In the absence of photoreceptors and their nuclei, we observed 49% greater imaged detail (cut-off chosen at 50% residual contrast, Figure 1B3). Photoreceptor nuclei contain highly compacted and molecularly dense DNA with significant light-scattering potential (Drezek et al., 2003; Marina et al., 2012; Mourant et al., 2000), while photoreceptor segments have been described as image-preserving waveguides (Enoch, 1961). These findings suggest that light propagation in the mouse retina is significantly impacted, if not dominated, by the highly abundant rod nuclei of the ONL.

We then asked whether retinal cell somata are optically specialized with distinct light-scattering properties. We compared the light scattering by different cell types using high-throughput FACS (Feodorova et al., 2015) measurements. The suspensions of cells or papain-digested retinae were used to measure the cellular light scattering in the far-field using a commercial FACS set up. These measurements revealed that cells isolated from the mouse retina known to typically consist of ~80% rod photoreceptor cells (Hughes et al., 2017), scatter substantially less light than neurons of the brain and cultured neuroblastoma cells (Figure 1C). This trend is seen for forward-scattered light (measured in a narrow range around 0°) but is even more pronounced for side scattering (measured around 90 degrees, see supplementary methods for details), which reflects subcellular heterogeneity. Using forward scattering as a measure of cell size indicates that side scattering normalized by volume (volume-specific light scattering) is also noticeably lower in retinal cells (Figure 1C, inset). This suggests that retinal cells are indeed optically specialized, as they scatter less light for a given size. This unique property for the rod cells could stem from the unusually dense packing of the heterochromatin in the centre of their nuclei, which notably even excludes free GFP molecules (Figure 1—figure supplement 1B).

To determine when the low sideward light scattering characteristic of retinal nuclei emerges, we compared the scattering profile of retinal nuclei in P25 WT pups and WT adult (12 weeks) mice. We found little or no difference between forward light scattering (Figure 1D–E), as predicted by earlier models (Błaszczak et al., 2014; Kreysing et al., 2010; Nagelberg et al., 2017). In stark contrast however, sideward scattering, with a strong potential to diminish image contrast, was significantly reduced in adult retinal nuclei compared to the intermediate developmental stage (Figure 1F). Quantitative analysis of sorted nuclei from P25 retinae further revealed a monotonic relation between chromocenter number and sideward scattering signal (Figure 1G). In particular, those nuclei with the lowest number of chromocenters were found to scatter the least amount of light. In support of this experimental quantification, a wave-optical Mie model of light-scattering by refractive chromocenters closely reproduced the trend of light scattering reduction with chromocenter fusion (Figure 1H).

**Figure 2.**
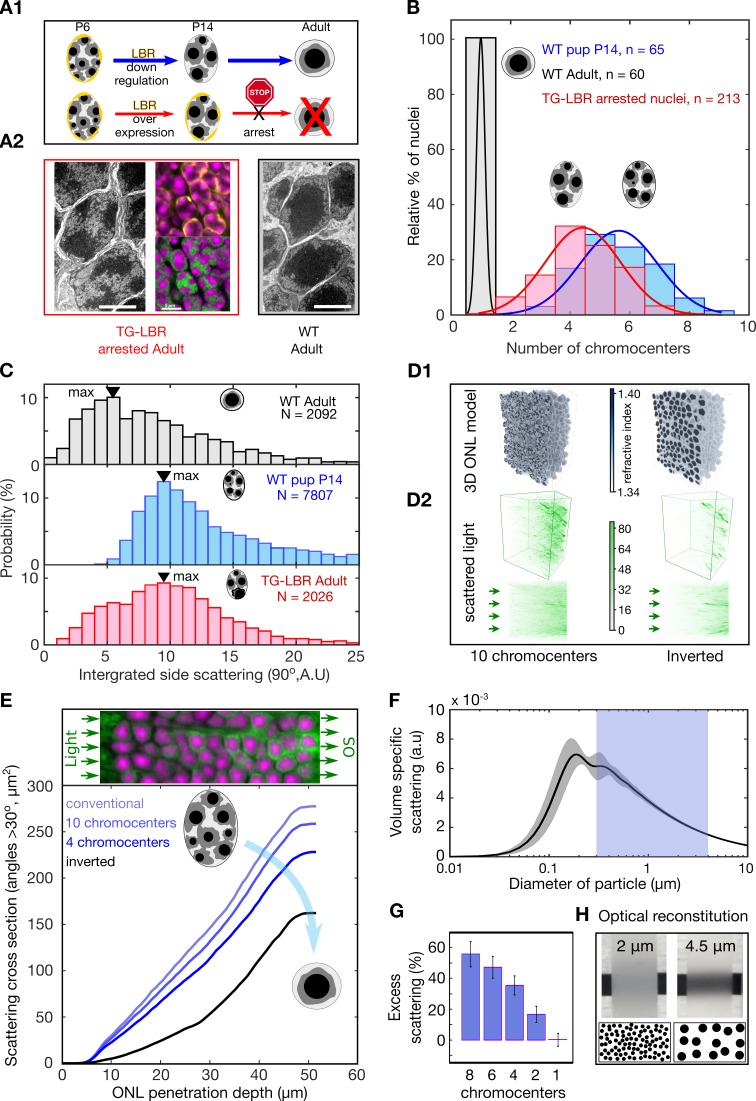
Developmental arrest of chromocenter fusion increases light scattering from rod nuclei in measurements and tissue simulations. (**A1**) Schematic of the normal rod nuclear WT development and inversion arrested nuclei by LBR overexpression. (**A2**) EM images illustrating different electron densities in the euchromatic and heterochromatic phase underlying their refractive index (RI) differences (scalebar 5 μm). (mid-top) Immunostaining of overexpressed of LBR tethers (yellow), and high-density heterochromatin (DAPI, magenta). (mid- bottom) Heterochromatic chromocenters (DAPI, magenta) and euchromatin (H4K5ac, green) (**B**) Chromocenter number distribution in LBR overexpressing rod nuclei is drastically different from WT mice, and similar to a developing WT pup (P14). (**C**) Side scattering assessed by FACS for TG-LBR retina nuclei is higher than that of WT nuclei and comparable to that of a WT P14 nuclei with similar chromocenter numbers. Note the shift of peak value upon LBR overexpression. (**D1**) 3d RI distribution mapped onto anatomically faithful volumetric ONL images. WT inverted architecture (right, top) and early developmental state (left) (simulation). (**D2**) (top) Differential simulations of light propagation in the ONL, using same positions and shapes of about 1750 nuclei, but varying chromatin distributions. (bottom) Maximum projection illustrating greater proportions of scattered light (angles > 30 deg) in the ONL with multiple chromo-centered nuclei. (**E**) Quantitative analysis of this data. (**F**) Angle weighted volume-specific scattering strength for nuclei models evaluated by Mie scattering theory. (**G**) Excess scattering occurring in multi-chromocenter nuclei models. (**H**) Chromocenters scattering reconstituted in an emulsion of silica spheres in glycerol-water mixture. Pictograms reflect accurate number ratio of spheres.

To establish whether rod nuclear inversion is required to cause the developmental reduction in light scattering, we used a transgenic mouse model (TG-LBR) in which heterochromatin remains anchored at the lamina which in turn prevents the complete fusion of chromocenters (Figure 2A1, A2 and B, Figure 1—figure supplement 2, Figure 1—figure supplement 1; Figure 2—figure supplement 1) (Solovei et al., 2013). FACS experiments of nuclei from TG-LBR retinae, in which > 70% of the nuclei are successfully arrested (Figure 2—figure supplement 2), revealed significantly increased light scattering (Figure 2C, Figure 1F). Specifically, the global maximum of the side-scattering was re-located precisely to the position that is characteristic of nuclei isolated from WT pups at P14, which possess a similar number of chromocenters as inversion arrested nuclei (compare Figure 2B,C, Figure 1F,G). Because inhibition of chromocenter fusion leads to specific increase in scattering, we conclude that the reduction of light scattering with chromocenter number is causal.

**Figure 2—figure supplement 1.**
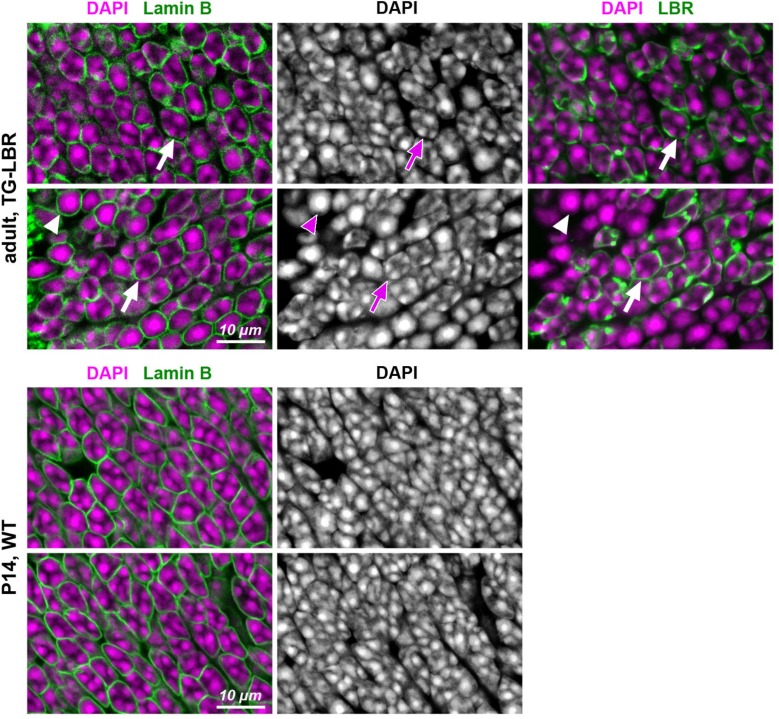
Distribution of chromocenters in nuclei of adult rods transgenitically expressing LBR in comparison to nuclei of P14 WT rods. Exemplified areas of ONL in adult TG-LBR (upper raw) and P14 WT (bottom raw) retinas. Note that only a proportion of rods in transgenic retina express LBR (see also Figure 1—figure supplement 2). Nuclei of rods expressing LBR exhibit conventional chromatin arrangement with chromocenters adjacent to the nuclear envelope (arrows); rod nuclei not expressing LBR remain inverted with one central chromocenter (arrowheads). P14 WT rod nuclei have still conventional nuclei, although exhibit signs of ongoing inversion with massive chromocenter fusion. Immunostaining for lamin B is used to outline nuclear border (green, left panel); immunostaining for LBR is used to highlight transgenic rods (green, right panel); nuclei are counterstained with DAPI (magenta). Single confocal sections.

**Figure 2—figure supplement 2.**
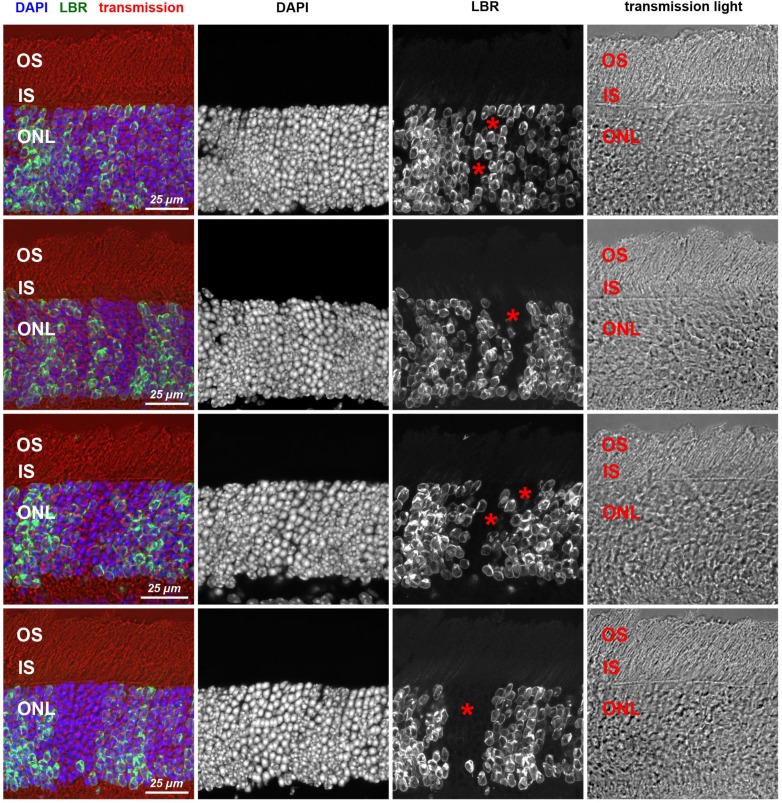
Transgenic expression of LBR does not influence rod photoreceptor structure. Retina areas with LBR-positive and LBR-negative (asterisks) rod clones demonstrating unaltered stratification. The three photoreceptor layers, represented by photoreceptor perikarya (ONL), cytoplasm of inner segments (IS) and dendritic ends of outer segment (OS), are preserved in TG-LBR retina. Immunostaining of LBR (green); nuclear counterstain with DAPI (blue). Maximum intensity projections of 5–7 µm confocal stacks.

### Improved retinal contrast transmission

Next, we asked how nuclear substructure could affect the optical properties of the ONL. We first approached this via a simulation that built on recent advances in computational optics (Weigert et al., 2018). This allowed us to specifically change nuclear architecture, while leaving all other parameters, including the morphology and relative positioning of about 1750 two-photon mapped nuclei, unchanged (Figure 2D1, Videos 1 and 2) (Supplementary Methods).

**Video 1.** 2 photon volumetric image of WT mouse retina.

**Video 2.** 3D morphological models of ONL RI distribution used in light propagation simulations.

These simulations suggest that especially the large-angle scattering (cumulative scattering signal at angles > 30 deg) monotonically decreases when 10 chromocenters successfully fuse into one (Figure 2D2 and E). Physically, this effect of reduced scattering can be explained by a reduction of volume-specific scattering for weak scatterers in the size regime slightly above one wavelength of light, similar to scattering reduction techniques proposed for transparent sea animals (Johnsen, 2012) (Figure 2F,G). Furthermore, a minimal optical ONL model reconstituted from suspended beads of different size but same volume fraction (Supplementary Methods) illustrates how a decreased geometric scattering cross section after fusion leads to reduced scattering-induced veil that helps to prevent contrast losses (Figure 2H and inset). Taken together these data suggest that nuclear inversion might serve to preserve contrast in retinal transmitted images.

To experimentally quantify the optical quality of the retina with respect to nuclear architecture, we applied the concept of the modulation transfer function (MTF), a standard way to assess image quality of optical instruments (Boreman, 2001). Specifically, MTF indicates how much contrast is maintained in images of increasingly finer sinusoidal stripes (Figure 3—figure supplement 1B,C). We therefore devised an automated optical setup (Figure 3—figure supplement 1A) that allowed us to project video sequences of demagnified sinusoidal stripe patterns through freshly excised retinae and assess the retinal transmitted images for contrast loss. This custom built set-up mimics the optics of the mouse eye, in particular its f-number (Schmucker and Schaeffel, 2004), while circumventing changes of the optical apparatus in-vivo (Figure 3—figure supplement 1, Materials and methods).

**Figure 3.**
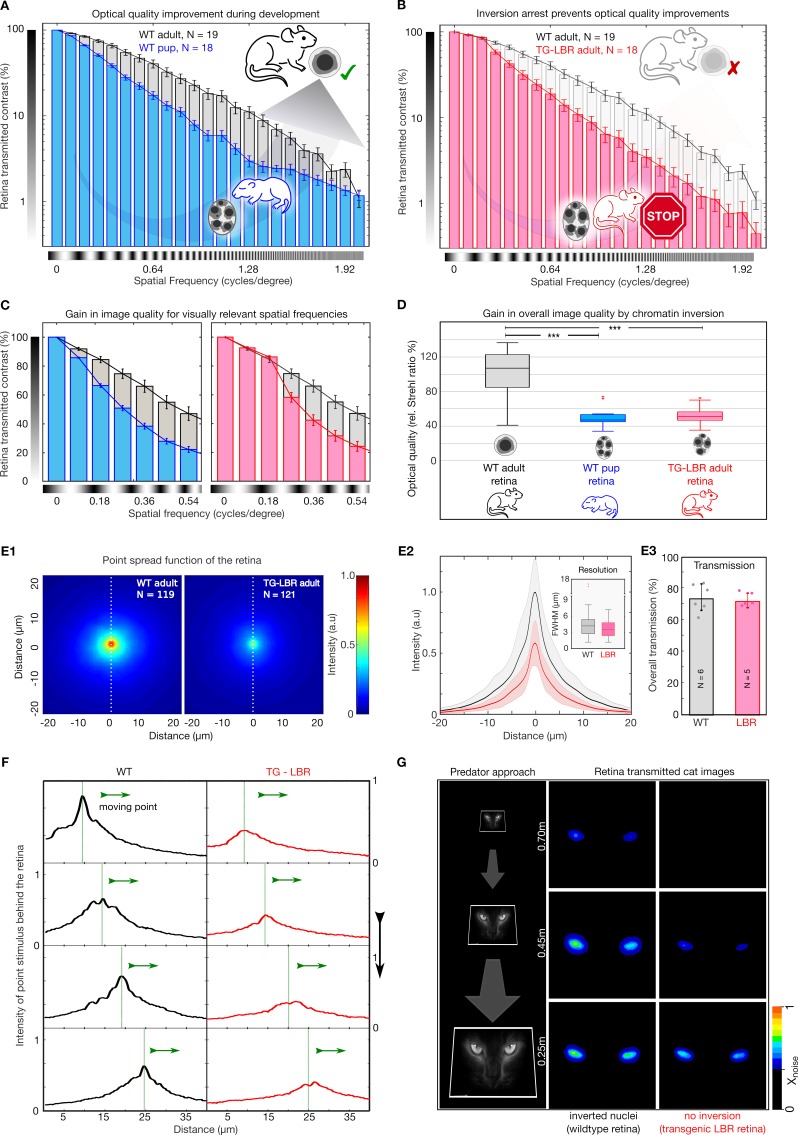
Nuclear inversion improves retinal contrast transmission characteristics. (**A**) Retinal contrast transmission increases during developmental stages of nuclear inversion, as experimentally revealed by measurements of retina-transmitted sinusoidal stripe patterns (modulation transfer functions). Developmental stage P12-14 (N = 18), compared to wildtype adult (N = 19 animals), note log scale. (**B**) These improvements in optical quality do not occur in retinae in which rod nuclei are transgenitically arrested in development and maintain 4–5 chromocenters. TG-LBR mouse (N = 18 animals) compared to WT reference (N = 19 animals), N = 1950 images in total. Mean + /- 95% CI. (**C**) Retinal contrast transmission at visually relevant spatial frequencies showcasing on an average ~49% and~37% better contrast transfer by the WT Adult retina (grey) in comparison to the WT-P14 pup (blue) and TG-LBR Adult (red) respectively. (**D**) The optical quality improvement of the retina (relative Strehl ratios), as caused by nuclear inversion, is two-fold (p=1.1880e-08 - WT adult vs WT pup, 3.4055e-08 - WT adult vs TG-LBR adult, 0.4761 - TG-LBR adult vs WT pup). (**E1**) Point spread function (PSF) for WT and LBR adult retinae by projection of 3 µm point light stimuli through the retina, N = 240 measurements in total six retinae. (**E2**) Intensity quantification along the white dotted line. Shaded region shows ±1 sd. Comparable resolution in transmitted images as assessed by the FWHM of the psf (inset). (**E3**) Near identical diffuse light transmission by both WT and TG-LBR retinae (n = 2 animals each, mean ± s.d.) (**F**) Intensity of a moving, retina-transmitted point stimuli for WT (black) and TG-LBR mouse (red). (**G**) Image-series of a cat approach as seen through the retina of mice, WT and transgenic genotype from various behaviorally relevant distances at the same vision limiting (arbitrarily chosen) signal to noise level. Consistent intensity differences of two or more color shades indicate significantly better predator detection potential for WT mice. Data magnified for clarity.

Strikingly, we found that wildtype retinae improve contrast transmission throughout terminal development, with adult retinae showing consistently elevated MTFs compared to intermediate developmental stages (P14) in which rod nuclei still possess around five chromocenters (Figure 3A). In contrast to many lens-based optical systems, retinal MTFs have a long tail with non-zero residual contrast despite an initial rapid loss of contrast (a characteristic of scattering-dominated optical systems). The monotonic decay of retina-transmitted contrast indicates scattering-induced veil, rather than a frequency cut-off to be the cause of contrast loss (Figure 3A,B Figure 3—figure supplement 2A–D). Collected from >1300 high resolution images, this data reveals that, similar to the lens (Tkatchenko et al., 2010), the retina matures towards increasing optical quality during latest developmental stages, with chromocenter fusion as a putative mechanism of veil reduction.

**Figure 3—figure supplement 1.**
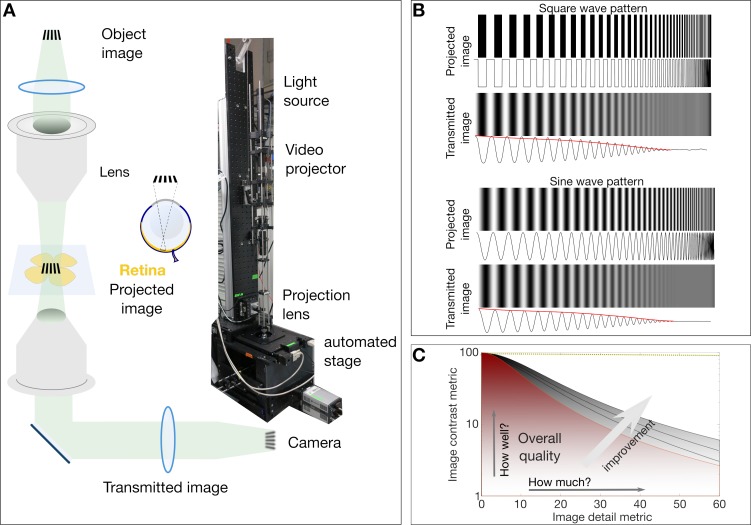
Simplified schematic of the custom micro-projection setup and the concept of modulation transfer function. (**A**) Simplified schematic and photo of the micro projection setup indicating the image object. The projecting lens functions like the biological eye (same NA = 0.45) to project the image on to the retina. The transmitted image is collected by a second lens and recorded on a camera. (**B**) The loss of contrast inherent to imaging systems (compare contrasts and intensity signals in projected and transmitted images). The envelope of the gradually degreasing signal (shown in red) is essentially the transfer function of the imaging system. The study of the contrast at various image details and fineness results in the MTF curves as shown in (**C**). (**C**) A typical MTF plot of image contrast vs image detail parameters. In this depicted case the overall image quality is proportional to the area under the curve that can yield the Strehl Ratio (a combined metric of how well and how much of the image is visible).

**Figure 3—figure supplement 2.**
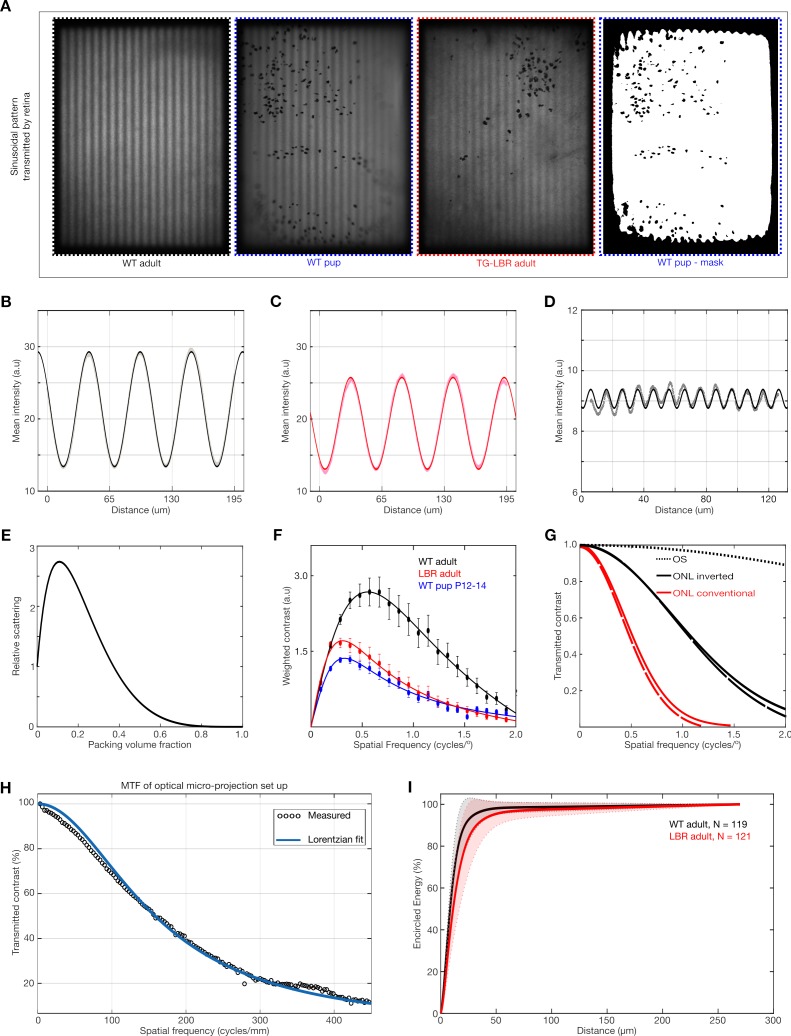
Modulation Transfer Function and its relation to light scattering and visual perception. (**A**) Representative images of sinusoidal patterns transmitted by the different retinae and a mask image (far right) illustrating the ROI used for contrast analysis. (**B, C**) Fitting of pure sinusoidal curves to the measured intensity in a WT and TG-LBR mouse retina at 0.1cyc/deg. The greater transmission of contrast is evident from the amplitude of the sine waves in the WT retina. (**D**) Illustration of robustness of the sine curve fitting for very low residual contrast (~3%) and very fine image details. (**E**) Dependent scattering effects due to close packing of scatterers for various volume fractions. (Calculations based on models described in literature). (**F**) Frequency weighted contrast transmission curves for the evaluation of the Strehl Ratio. (**G**) Estimates of comparison of MTF from modelling and simulation of light scattering by outer nuclear layer (ONL) and outer segments (OS) illustrating the dominant effect of the packed nuclei as opposed to the outer segments. (**H**) Modulation transfer function of the optical micro-projection set up. (**I**) Encircled energy (EE) plot. EE under the retinal PSF converges to the same value for both WT (black) and TG-LBR retinae (red) Shaded regions show standard deviation. N = 240 measurements in total six retinae.

Next, we asked if developmental improvements in contrast transmission of the retina are indeed caused by chromocenter fusion. For this we used mice in which LBR-overexpression largely arrested chromocenter fusion, resulting in an elevated number of chromocenters in the adult animal, similar to P14 WT (Figure 2A2, B), without displaying any effect on other morphological characteristics (Figure 2—figure supplement 2). Strikingly, repeating MTF measurements on adult retinae of this inversion arrested mouse model (TG-LBR), we find near identical contrast attenuation characteristics as in the developing retina (compare Figure 3A and B). Thus, developmental improvements of retinal contrast transmission are indeed mediated by the inversion of rod nuclei. Notably, when we focus on the retinal transmission data within the spatial frequency regime that is relevant for mouse vision (Alam et al., 2015; Prusky et al., 2004; Prusky et al., 2000), (Figure 3C) it can readily be seen that the contrast transmission is up to 33% greater in WT compared to TG-LBR at frequencies ~ 0.28 cycles/deg. Equally, the contrast transmission in this behaviorally relevant regime also increases up to 45% in WT adult compared to the pups.

Frequently, the quality of image-forming optical systems is reported as a single parameter value called the Strehl ratio (Thibos et al., 2004). Since our image projection setup closely mimics the mouse eye, it allows meaningful comparisons of the Strehl ratios of retinae, by comparing the volumes under MTF curves. With regards to our MTF measurements, we that find the Strehl ratio (computed using the measurements in the spatial frequency range of 0–2 cycles/deg) of a fully developed retina is increased 2.00 ± 0.15 fold compared to that of pups (P14) in which chromocenters fusion was not completed, and similarly 1.91 ± 0.14 fold (ratio of means ± SEM) improved compared to TG-LBR adult retinae (p=3.4055e-08) in which chromocenter fusion was deliberately arrested (Figure 3D).

Since the Strehl ratio makes predictions for the peak intensity of a tissue-transmitted point stimulus, we analysed the effect of micro-projecting a point-like stimulus through the mouse retina (diameter here ~3μm, measurement constrained by outer segment spacing). We found that the resulting image at the back of the WT retina had a near two-fold (1.79 ± 0.38, mean ± SD) higher peak intensity compared to the TG-LBR retina (Figure 3 E1 & E2, N = 119, N = 121, measured in at a total of 6 animals). The measured resolution based on full-width half maximum (FWHM) of the PSF, however, did not show any differences (4.32 ± 2.38 μm, 3.75 ± 2.01 μm, mean ± SD for WT and TG-LBR retinae respectively, Figure 3 E2), especially no changes that could physiologically impact acuity, which is known to be significantly lower in mouse. In addition to independently corroborating our MTF measurements, these results emphasize that nuclear inversion enhances contrast transmission through the retina but is unlikely to benefit acuity. From a mechanistic point of view, these measurements indicate that contrast is lost due to the generation of image veil from side scattering, which overcasts attenuated, but otherwise unchanged signals. Accordingly, when comparing the integrated absolute transmission through rhodopsin-bleached retinae in dedicated experiments (Supplementary methods), we found near identical transmission values for WT and inversion arrested retinae (Figure 3 E3, T_WT_74 ± 8%, T_LBR_ = 72 ± 5%, mean ± SD), which emphasizes that despite differential image signal, the overall photon arrival at the photoreceptor outer segments, remains unchanged.

An advantage of improved retinal contrast transmission is suggested when following the motion of individual (non-averaged) light stimuli that appear at considerably higher signal-to-noise ratios (Figure 3F) at the outer segments level. A putative visual advantage to appropriately scaled real-life examples, such as images of an approaching cat micro-projected through a mouse retina is illustrated in Figure 3G. Nuclear inversion results in cat images becoming visible considerably earlier compared to mice that lack nuclear inversion (0.70 vs 0.45 meters, at a given arbitrary noise threshold). These results suggest that nuclear inversion may offer enhanced visual competence that originates from improved contrast preservation in retinal images. More objective and established methodologies to test the impact of nuclear inversion for actual behavior is addressed in the next section.

### Improved contrast sensitivity

**Figure 4.**
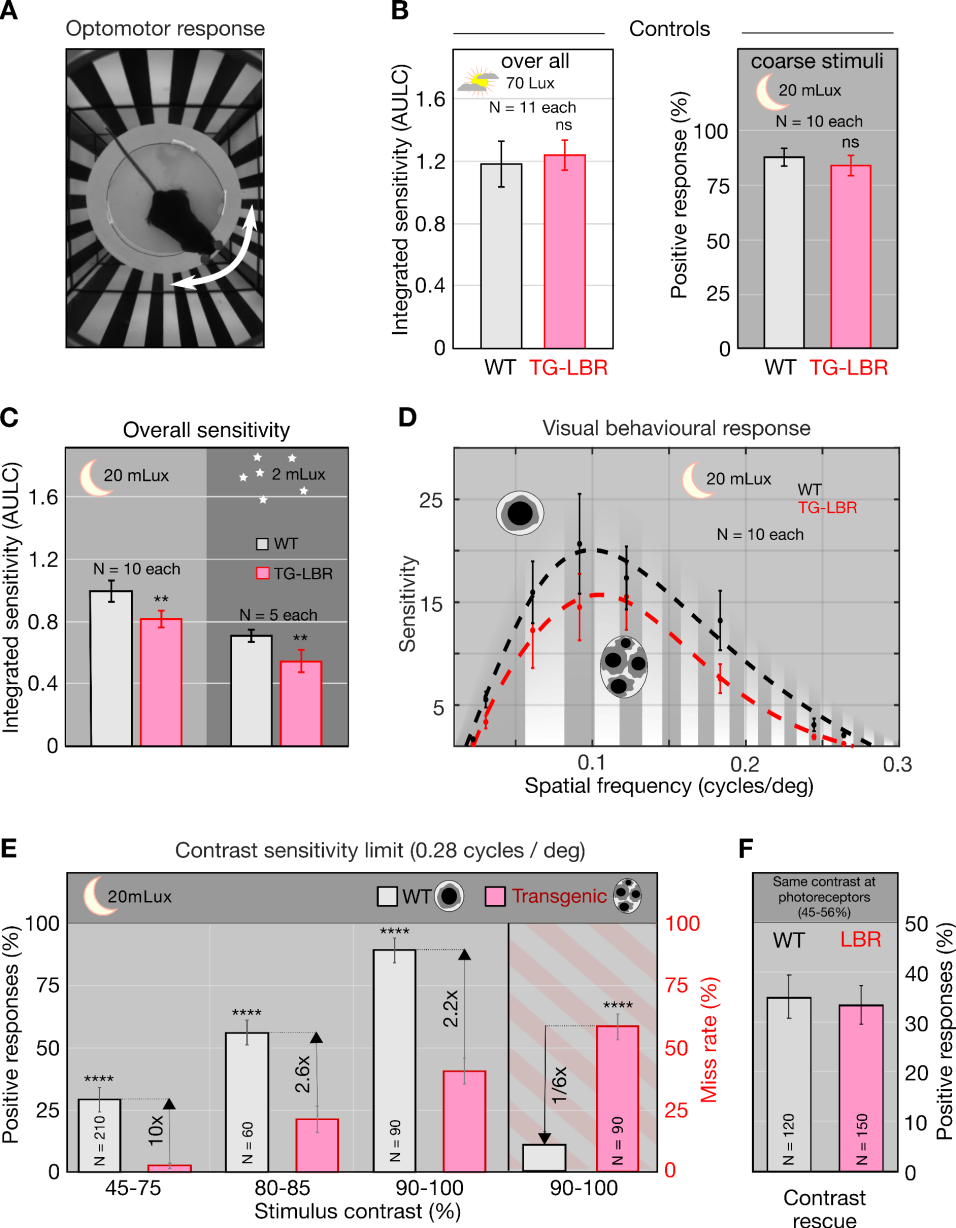
Nuclear inversion improves contrast sensitivity in the dark. (**A**) Illustration of the automated optomotor response experiment to assess the visual performance of mice, shown a 0.06 cycles/deg. (**B**) Photopic control condition and scotopic coarse stimulus (0.06cycles/deg) control showing no significant difference between WT and TG-LBR mice (p=0.5307, p=0.2842, t-test, Chi-square test). (**C**) Under scotopic conditions (20 and 2 mLux) the overall sensitivity of the WT mice is 22% and 29% higher than TG-LBR mice (area under log contrast sensitivity curve, AULC) mean+/-95% CI, (p=0.00081, 0.0047, two sample t-test). (**D**) Contrast sensitivity curves evaluated at 20mLux light intensity. Significant differences appear at angular sizes above 0.15 cycles/deg (p=0.038, two sample t-test). (**E**) Behavior differences are strongest for stimuli close to the visual threshold. Here the mice in possession of the inverted rod nuclei (WT) possess an up to 10 times higher sensitivity at intermediate contrasts (29% vs 3% correct response in 45–75% contrast range), and a six times reduced risk to miss a motion stimulus at high contrasts (10 vs 59% failure in detection, 0.26–0.3 cycles/deg), (p<0.0001, Chi-square test) mean ± s.d. (**F**) Rescue experiment demonstrating sufficiency of improved retinal contrast transmission to explain improved sensitivity. Adjusting the level of contrast at the photoreceptor level (by pre-compensation of differential contrast loss) restores sensitivity of TG-LBR mice. N indicates number of individual trials of 10 animals together for each mouse type.

To determine whether the improved retinal contrast transmission translates into improved visual perception, we carried out behavioral tests using *Optomotor-reflex* measurements (Figure 4A, Video 3). Specifically, we used a fully automated mouse tracking and data analysis pipeline (OptoDrum, Striatech, Germany) (Benkner et al., 2013) to compare the contrast sensitivities of adult WT mice and those with arrested nuclear architecture (TG-LBR). Firstly, contrast sensitivity assessed by the animal’s ability to detect moving stripes, did not differ significantly (p=0.5307, two sample t test) between the two genotypes at photopic light condition (70 Lux – the typical brightness of monitor). Transgenic and WT animals showed comparable visual sensitivity, as quantified by the area under the log-contrast sensitivity curve (AULC, Figure 4B, left) (Villegas et al., 2002). As nuclear adaptation is strongly correlated with nocturnal lifestyle (Solovei et al., 2009; Solovei et al., 2013), we adapted this set-up to assess contrast sensitivity under scotopic light conditions. At 20 mLux, which is the range of brightness in moonlight (Kyba et al., 2017), we again found comparable responses for coarse stimuli (wide large contrast stripes) suggesting equally functional rod-based vision in TG-LBR and WT mice (Figure 4B, right) without noticeable differences in absolute sensitivity. Furthermore, mice deficient of rhodopsin (*Rho-/-*) (Humphries et al., 1997; Jaissle et al., 2001) confirmed that visual behavior under the displayed scotopic conditions fully relies on the functionality of the rod pathway (Figure 4—figure supplement 1E).

**Figure 4—figure supplement 1.**
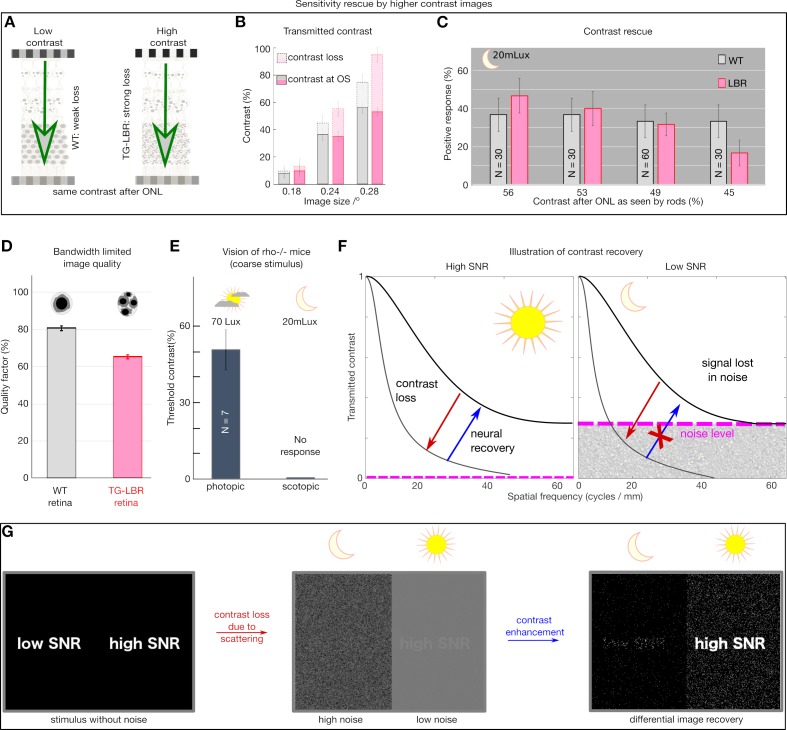
Retina transmitted contrast directly impacts visual behavior. (**A**) Illustration of the role of retina as an information filter and contrast loss buckets for WT and TG-LBR retina. (**B**) Stimuli contrast for OMR response presented such that after corresponding loss in the WT and TG-LBR retinae, the outer segments receive comparable contrast of ~9%, 36% and 54% at 0.18, 0.24, 0.28 cycles/deg respectively for transduction. (**C**) The visual response of the TG-LBR mouse is recovered to the level of WT mouse by a stimulus with higher input image contrast compensating for the greater loss due to its ONL with highly scattering non-inverted nuclei. (**D**) Estimates of image quality for the corresponding mice based on the MTF of WT and TG-LBR retina at relevant range of visually sensitive image details (0.15–0.36 cycles/degree). (**E**) Control to verify the scotopic illumination settings of the OKT setup, depicting no response for a rhodopsin KO mouse. (**F**) Day and night vision differ significantly in the signal to noise level (Warrant, 1999), and loss of signal below the noise floor is lost from an information point of view and cannot be recovered. (**G**) Illustration of the contrast recovery for stimulus under low and high noise conditions. The scattering induced contrast loss and image degradation in a noisy low light environment cannot be recovered by any contrast enhancement mechanism.

**Figure 4—figure supplement 2.**
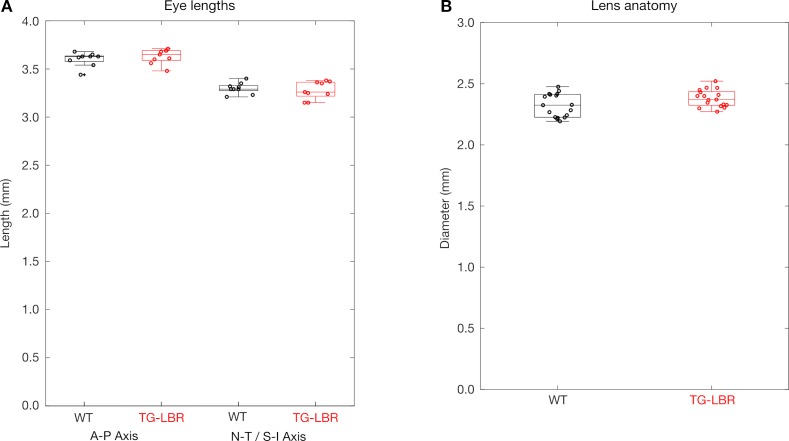
Ocular parameters of WT and TG-LBR mice are comparable. (**A**) The overall size of the eye as measured by length of the eye along the anterior - posterior (A–P) axis and the naso-temporal (N–T) or superior-inferior (S–I) axes (N = 9 eyes each) and (**B**) the measured diameter of the lens (N = 19 lens each) are comparable for WT and TG-LBR adult mice.

**Figure 4—figure supplement 3.**
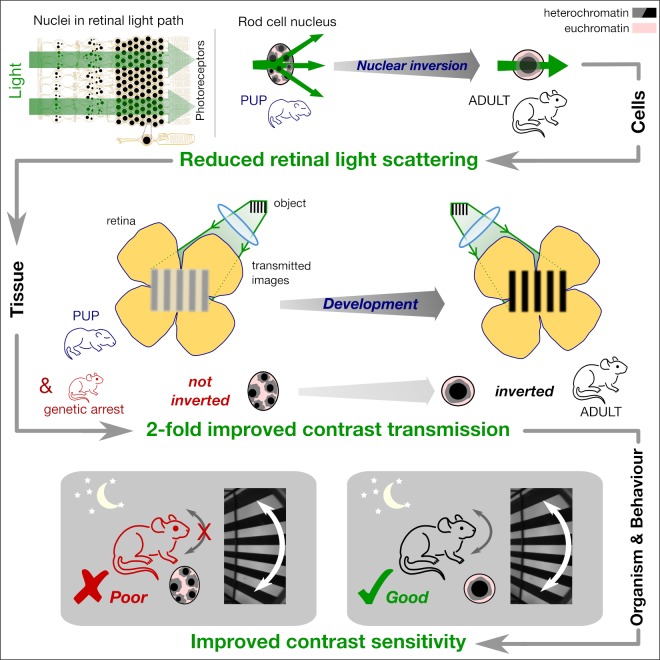
Model of nuclear adaptation enhancing nocturnal vision. The abundance of rod nuclei in the nocturnal mammalian retina presents a significant barrier to light. The fusion of heterochromatic chromocenters reduces the volume-specific scattering mainly at large angles. This results in a reduced light scattering at the tissue level leading lower scattering induced veil. Reduction in light scattering leads to a near two-fold improvement in the contrast transmission by the retina. Improved image quality transmitted by the retina finally enables greater contrast sensitivity exclusively at nocturnal conditions.

**Video 3.** Behaving mouse in an Optomotor response set up.

When required to detect finer stripes, WT and TG-LBR mice displayed significant differences in their visual performance, specifically in contrast sensitivity (Figure 4C). At 20mLux we observed an 18% greater AULC for WT mice compared with TG-LBR mice (p=0.00081). At even lower light intensities (2mLux, comparable to a starry night), the difference in AULC values was even greater (ratio 27%, p=0.0047) albeit at lower absolute sensitivities, which agrees with reported values for WT mice (Alam et al., 2015; Prusky et al., 2000; Prusky et al., 2004). The most significant differences in the contrast sensitivity occur above 0.15 cycles/degree (Figure 4D). Especially, in the regime close to the visual acuity (0.26–0.30 cycles/degree), WT mice show up to 10 times (p<0.0001) greater positive response rates at intermediate contrasts (Figure 4E) compared to mice with inversion arrested rod nuclei. Moreover, at 90–100% contrasts, where WT mice approach a maximum responsiveness, we observed a near 6-fold reduced risk to miss a stimulus for WT compared to TG-LBR mice (false negative rates 11% WT, 59% TG-LBR).

Finally, we asked whether reduced visual sensitivity of mice lacking the inverted nuclear architecture can be sufficiently explained by inferior contrast transmission of the retina. Direct comparison of behavioral sensitivity with the MTF curves showed that vision mostly occurs in regions in which retinal contrast transmission is higher than 50% and substantial differences in MTFs occur. Specifically, the 18–27% difference in contrast sensitivity goes together with a 26% higher Strehl ratio in WT retinae when evaluated in the relevant frequency regime (0–0.36 cycles/degree).

This suggests that at low light levels, contrast sensitivity may be directly limited by contrast transmission through the retina, and that a reduction of contrast sensitivity in mice with non-inverted rod nuclei may be explained by increased contrast losses in the retina. Moreover, we did not observe unexpected side effects from the LBR overexpression, at level of retinal (Figure 2—figure supplement 2), ocular or lens anatomy (Figure 4—figure supplement 2), and non-limiting rod vision was normal (Figure 4B (Left)). Nevertheless, it is clear that the complexity of the eye does not permit an exhaustive comparison of all parameters that could potentially be affected by LBR overexpression, including subtle concentration changes in molecules relevant for phototransduction. So how can one rule out the possibility that the loss of sensitivity in LBR overexpressing mice is due to a loss of image contrast, rather than unspecific side effects?

To show that increased contrast sensitivity in WT mice is due to the increased contrast transmission of the retina, we designed a rescue experiment logically equivalent to rescue experiments that show specificity of molecular interventions. Frequently, one excludes nonspecific side effects of a molecular knock-down by rescuing the phenotype via the addition of the protein of interest (if possible a pathway-specific variant of this protein). To show that sensitivity is lost due to retinal loss of contrast, we performed an optical rescue experiment. For this, we first confirmed that contrast transmission through the inner retina is a linear process, with contrasts at the photoreceptor levels being proportional to contrasts in projected images (Figure 4—figure supplement 1B,C). We then adjusted the displayed contrasts in optomotor measurements to pre-compensate for higher contrast losses in the TG-LBR retina while also conserving image intensities. Strikingly, we found that with equal image contrast at the level of the photoreceptor segments, visual competence of LBR mice was rescued and becomes near identical to that of WT mice (Figure 4F). Thus, improved retinal contrast transmission is indeed sufficient to explain increased contrast sensitivity in mice.

## Discussion

As an important determinant of fitness, animals evolved a wide range of visual adaptation to see in the dark (Nilsson, 2009; O'Carroll and Warrant, 2017; Thomas et al., 2017; Warrant and Nilsson, 2006; Warrant, 2017). Nocturnal vision is known to rely on highly efficient light capture, both at the level of the lens and photoreceptor outer segments, and often compromises spatio-temporal resolution by summation strategies of neuronal readout (Warrant, 1999; Warrant, 2017). Here we established nuclear inversion as a complementary strategy to maximize sensitivity under low light conditions. Centrally, we show that it is the direction into which light is scattered inside retinal tissue that translates into differential contrast sensitivity. Specifically, we find that the forward scattering characteristic of inverted nuclei (Kreysing et al., 2010; Solovei et al., 2009) mainly suppresses light scattering by nuclear substructure towards large angles, thus preventing image veil and contrast reduction resulting from it.

As mammalian eyes are evolutionarily multi-constrained systems, one could ask if nuclear inversion might also serve other functions beyond the improved contrast sensitivity that we have showed. Slightly reduced thickness of the ONL might translate into more efficient diffusion of nutrients, waste and signals. Similarly, the unusually large fraction of hetero-chromatin in rod photoreceptor cells (Wang et al., 2018), which might enable the small nuclear volume and/or more efficient fate-specific gene silencing (Becker et al., 2017; Hiler et al., 2015; Mattar et al., 2018; Wang et al., 2018), might hypothetically lead to architectural problems for the nucleus which could be circumvented by nuclear inversion. As an example, chromatin distribution is known to have the potential to modulate the mechanical properties of the nucleus (Kirby and Lammerding, 2018; Miroshnikova et al., 2017; Stephens et al., 2017) and LBR downregulation might even be required for shape changes enabling efficient packing of nuclei (Stephens et al., 2019). Lastly, although the size of the PSF is beyond the acuity limit of nocturnal vision, the increase in intensity of a point stimulus at the photoreceptor level could aid a thresholded or otherwise non-linear readout of rod cells, a long-standing hypothesis in the field of visual neuroscience (Barlow, 1956; Field and Rieke, 2002; Nelson, 2017) which was substantiated by the use of quantum-based single-photon sources (Tinsley et al., 2016).

While these additional functions of nuclear inversion currently remain speculations, it is worth reflecting about the relevance of the visual benefits demonstrated here for enhancing animal vision in general. Since our reported mechanism involves improvements in retinal image contrast rather than notable changes in photon transmission that could impact absolute sensitivity (Banks et al., 2015; Cronin et al., 2014; Nilsson, 2009; Warrant, 1999), one might ask why nuclear inversion as an adaptation is exclusive to nocturnal mammals. Wouldn’t improvements in retinal image contrast not also be beneficial for diurnal mammals? Firstly, the larger spacing of photoreceptor segments in the diurnal retina significantly reduces ONL thickness (Solovei et al., 2009; Sterling and Laughlin, 2015; Werner and Chalupa, 2004; Williams and Moody, 2004) and thereby the risk of scattering induced veil and loss of image contrast. Furthermore, as is well known from photography, shot-noise that accounts for image granularity (Barlow, 1956; de Vries, 1943; Rose, 1948) becomes less of a problem with increasing light levels. Million-fold higher light intensities during the day imply a higher safety margin from this noise floor (Warrant, 1999), (Figure 4—figure supplement 1F,G), as required for neural mechanisms of contrast enhancement to function (Artal et al., 2004; Flevaris and Murray, 2015; Hess et al., 1998; Shevell et al., 1992). Such compensatory mechanisms are also likely to explain why no behavioral differences are observed at elevated intensities and why augmented vision becomes pronounced only at low light levels. Last, but not least, our measurements show that, although nuclear inversion improves retinal contrast transmission via reduced image veil, resolution, the limiting factor for high acuity diurnal vision, remains largely unaffected. Besides a reduced need for inverted photoceptor nuclei in diurnal mammals, reduced efficiency of canonical DNA repair mechanisms (Frohns et al., 2014) in highly condensed chromocenters, could mean a significant disadvantage in the diurnal retina and susceptibility to stress and degeneration (Boudard et al., 2011; Dyer, 2016), could also mean a significantly higher cost for inverted nuclei in diurnal species, as their retinae are intrinsically strongly exposed to high-energy, ultra-violet photons.

In conclusion, we showed that rod nuclear inversion is necessary and sufficient to explain optically enhanced contrast sensitivity in mice (Figure 4—figure supplement 3). Our work thereby adds functional significance to a prominent exception of nuclear organization and establishes retinal contrast transmission as a new determinant of mammalian fitness.

## Materials and methods

**Key resources table:** 

Reagent type (species) or resource	Designation	Source or reference	Identifiers	Additional information
Strain, strain background (*M. musculus*)	C57BL/6NRj, (WT)	Janvier Labs		Colony maintained at biomedical facility of MPI-CBG
Strain, strain background (*M. musculus*)	Tg(Nrl-EGFP)	Kind Gift from Jung-Woong Kim (Anand Swaroop laboratory, Ophthalmology and Visual Sciences, University of Michigan, Ann Arbor).		(Akimoto et al., 2006)
Strain, strain background (*M. musculus*)	ROSA26-eGFP-DTA	Kind gift from Dr. Dieter Saur Klinikum rechts der Isar, Technische Universität München		(Ivanova et al., 2005)
Strain, strain background (*M. musculus*)	TG-LBR (Nrl-*Lbr*)	This paper, Dr. Irina Solovei, LMU Munich (Solovei et al., 2013)	biomedical facility of MPI-CBG	Materials and methods Tissue preparation for optical characterization and Flow cytometry
Strain, strain background (*M. musculus*)	Rd1/Cpfl1-KO	Ader Lab, CRTD Dresden, TU Dresden	Animal facility of CRTD	Materials and methods
Strain, strain background (*M. musculus*)	Rho-/-	(Humphries et al., 1997; Jaissle et al., 2001)	Animal facility of CRTD	Materials and methods
Cell line (*M. musculus*)	Neuro-2a (Neuroblast cells)	DSMZ	ACC-148; RRID: CVCL_0470	Cell line maintained as per ATCC recommendations
Biological sample (*M. musculus*)	Retina	This paper	biomedical facility of MPI-CBG,Animal facility of CRTD	Materials and methods Tissue preparation for optical characterization and Flow cytometry
Biological sample (*M. musculus*)	Brain sections	This paper	biomedical facility of MPI-CBG	Materials and methods Flow cytometry
Antibody	anti-lamin B (Goat, polyclonal)	Santa Cruz	SC-6217, RRID: AB_648158	IF (1:50)
Antibody	anti-LBR (Guinea pig, polyclonal)	Kind gift from Dr.H.Herrmann (DKFZ, Heidelberg)		IF (1:50)
Antibody	anti- H4K5ac (Mouse monoclonal)	Kind gift from Dr.H.Kimura (Tokyo Institute of Technology, Yokohama)	Clone 4A7	IF (1:100)
Sequence-based reagent	FISH Probes	This paper, Refer to methods for primer sequences.	PCR primers	(Solovei, 2010; Solovei et al., 2007).
Commercial assay or kit	Papain dissociation system kit	Worthington Biochemical Corporation	PDS LK003150	
Software, algorithm	Mie calculations	MATLAB Script	omlc-mie	(Mätzler, 2002)
Software, algorithm	Calculation of MTF	MATLAB	https://de.mathworks.com/products/matlab.html	Version 2017b, 2018b,
Software, algorithm	Retinal light propagation	biobeam	biobeam	(Weigert et al., 2018)
Software, algorithm	SPSS	IBM, SPSS	ibm-spss	Version 25
Software, algorithm	Optodrum	Striatech GmbH	Striatech	
Other	Alexa555	Invitrogen	A31570; RRID: AB_2536180	Fluorescent dyes
Other	Alexa 488	Invitrogen	A21202; RRID: AB_141607	Fluorescent dyes
Other	Hoechst	Thermo Scientific	33342	Fluorescent dyes
Other	Vectashield	Vector Laboratories, Inc, USA	Cat. No. H-1000–10	Antifade media
Other	Aqua Poly-Mount	Polysciences, Inc, USA	Cat. No. 18606–20	Antifade media
Other	FACS tubes	Corning Inc, USA	REF 352054	Falcon round bottom polystyrene
Other	Research Beads	BD Biosciences	655050	BD FACSDiva CS and T
Other	Silica beads	Whitehouse Scientific	MSS002, MSS004a	
Other	ND-1.2 filter	Rosco Laboratories Inc	e-color+ #299,	

### Retina sampling and preparation of cryo-sections

Wild type retinas were sampled from C57/BL6 mice. Eye balls of Nrl-GFP mice (Akimoto et al., 2006) were kindly provided by Jung-Woong Kim (Anand Swaroop laboratory, Ophthalmology and Visual Sciences, University of Michigan, Ann Arbor). Tissues from ROSA26-eGFP-DTA mice (Ivanova et al., 2005) were kindly provided by Dieter Saur (Klinikum rechts der Isar, Technische Universität München). The Rd1/Cpfl1-KO mice were maintained in the Animal facility of the CRTD, Dresden. Preparation of retina cryosections was performed according to protocol described earlier (Eberhart et al., 2013; Eberhart et al., 2012; Solovei, 2010). The enucleated eye balls were shortly washed with EtOH, punched with gauge 23 needle in the equatorial plane and fixed with 4% paraformaldehyde (PFA) (Carl Roth GmbH, Germany) in phosphate-buffered saline (PBS) solution for 3 hr. After fixation, samples were washed with PBS 3x 1 hr each, incubated in 10%, 20% and 30% sucrose in PBS at 4°C for 30 min in each concentration and left in 30% sucrose for overnight. The eyeballs were cut equatorially to remove the anterior parts, including cornea, lens and the vitreous, and eye cups were placed in a mold (Peel-A-Way Disposable Embedding Molds, Polysciences Inc) filled with tissue freezing medium (Jung tissue freezing medium, Leica Microsystems). Frozen blocks were prepared by either immersion of molds with tissues in freezing medium in a 100% ethanol bath precooled to −80°C, or by placing into a container filled with precooled to −70°C 2-methylbutane. After freezing, blocks were transferred to dry ice and then stored at −80°C. Cryosections with thickness of 14–20 μm were prepared using Leica Cryostat (Leica Microsystems) and collected on SuperFrost (Super Frost Ultra Plus, Roth, Germany) or StarFrost microscopic slides (StarFrost, Kisker Biotech GmbH and Co). After cutting, sections were immediately frozen and stored in at −80°C until use.

### Immunostaining

Immunostaining was performed according to the protocol described in detail earlier (Eberhart et al., 2012; Eberhart et al., 2013). Prior to immunostaining, slides with cryosections were removed from −80°C freezer, allowed to thaw and dry at room temperature (RT) for 30 min and then re-hydrated in 10 mM sodium citrate buffer for 5 min. For the antigen retrieval, slides were transferred to a preheated to +80°C 10 mM sodium citrate buffer either for 5 min (H4K5ac) or for 25 min (lamin B and LBR staining). After brief rinsing in PBS at RT, slides were incubated with 0.5% Triton X100/PBS for 1 hr, and once more rinsed in PBS before application of antibodies. Primary and secondary antibodies were diluted in blocking solution [PBS with 0.1% Triton-X100, 1% bovine serum albumin (ICN Biomedicals GmbH) and 0.1% Saponin (SERVA)]. Incubation with antibodies was performed for 12–14 hr under glass chambers in humid dark boxes (Solovei, 2010; Solovei et al., 2007). Washings after incubation with antibodies were performed with PBS/0.05%Triton X-100, 3x 30 min at 37°C. Primary antibodies included anti-lamin B (Santa Cruz, SC-6217), anti-LBR (lamin B receptor; kindly donated by Harald Herrmann, German Cancer Research Center, Heidelberg), anti-H4K5ac (kindly donated by Hiroshi Kimura, Tokyo Institute of Technology, Yokohama). Secondary antibodies were anti-mouse IgG conjugated to Alexa555 (A31570, Invitrogen) and Alexa488 (A21202, Invitrogen). Nuclei were counterstained with DAPI or Hoechst added to the secondary antibody solution. After staining, the sections were mounted under a coverslip with Vectashield (Vector Laboratories, Inc, Burlingame, CA, USA) or Aqua Poly-Mount (Polysciences, Inc, USA) antifade media and sealed with nail polish.

For microscopic analysis of FACS sorted retinal nuclei, sorted nuclei were fixed with 4% PFA in PBS for 10 mins, stained with Hoechst 33342, washed 2x with PBS and mounted on slides under coverslips in antifade medium (see below). The imaging was performed on a confocal microscopy (Zeiss LSM 700 inverted) using a Zeiss 64x 1.4 oil objective.

### FISH

FISH on cryosections was performed as described earlier (Solovei, 2010; Solovei et al., 2007). Probes for LINE, B1 and major satellite repeat (MSR) were generated by PCR using the following primers:

5’-GCCTCAGAACTGAACAAAGA and 5’-GCTCATAATGTTGTTCCACCT for LINE1;

5’-CACGCCTGTAATCCCAGC and 5’-AGACAGGGTTTCTCTGTA for B1;

5’-GCGAGAAAACTGAAAATCAC and 5’-TCAAGTCGTCAAGTGGATG for MSR.

Probes were dissolved in hybridization mixture (50% formamide, 10% dextran sulfate, 1xSSC) at a concentration of 10–20 ng/μl and hybridized to sections of mouse retina for 2 days. Post-hybridization washes included 2xSSC at +37°C (3x 30 min) and 0.1xSSC at +61°C (10 min). Sections were counterstained with DAPI and mounted as after immunostaining (see above).

### Microscopy and image analysis

Single optical sections or stacks of optical sections were collected using either Zeiss LSM 700 or Leica TCS SP5 confocal microscopes equipped with Plan Apo 63x/1.4 NA oil immersion objective and lasers with excitation lines 405, 488, and 561 nm. Dedicated plugins in the ImageJ (Schindelin et al., 2012) program were used to compensate for axial chromatic shift between fluorochromes in confocal stacks, to create RGB stacks/images, and to arrange optical sections into galleries (Ronneberger et al., 2008)

To estimate the proportion of rods expressing LBR in retinas from TG-LBR mice, four stained cryosections from two homozygous mice were imaged. Not less than 12 image fields with pixel size of 100 nm were collected through each section. Scoring of LBR-positive and negative rods was performed in ImageJ using Cell Counter plugin. Number of chromocenters in TG-LBR rods and P14 WT pups was estimated in confocal stacks through retinas after FISH with major satellite repeat and lamin B immunostaining. Scoring of chromocenters in 210 and 65 nuclei of transgenic and P14 rods, respectively, was performed manually using ImageJ.

### Electron microscopy

For electron microscopy, eyes of adult WT and TG-LBR mice were fixed by cardiac perfusion with a mixture of 2% paraformaldehyde and 2.5% glutaraldehyde in 0.1M cacodilate buffer for 5 min. After eye enucleation, the eye-balls were further fixed in the same fixative for 1 hr and then postfixed with OsO_4_ in cacodilate buffer for 1.5 hr. Ultra-thin sections were stained with uranyl acetate and Reynolds lead citrate. Images were recorded with a megaview III camera (SIS) attached to a Philips EM 208 transmission electron microscope (FEI) operated at 70 keV.

### Flow cytometry

FACS scattering analysis of retinal cells and sorts according to light scattering profiles were performed based on previously published methodologies (Feodorova et al., 2015). The Flow cytometric analysis was performed using FACS Aria Fusion (BD Biosciences) equipped with 488 nm laser and a 70 µm nozzle. For performance tracking and to ensure stability of the scattering signal, calibration beads from BD biosciences (BD FACSDiva CS and T Research Beads, 655050) were used. The scattering signal height vs width was then used to gate for singlet cell populations. Cell aggregates and debris were excluded for the data analysis. The papain dissociation system kit from Worthington Biochemical Corporation was used to digest the tissues. All the solutions for the digestion were prepared according to the manufacturer’s recommendation. The retinae from adult mice were gently and quickly isolated from enucleated fresh unfixed mouse eyeballs. 250 µl Papain digestion solution contained in a 2 ml Eppendorf tube was equilibrated (for 15–20 min) in 5% CO2. Two to four retinae were transferred to the equilibrated solution and incubated in a thermomixer at 37°C at 700 rpm. The tubes were periodically checked by visual inspection to ensure proper dissolution of the tissue. After 15–20 mins of incubation, the digest was added to a tube containing 15 µl DNAase-EBSS. The mixture was then mechanically agitated by pipetting the solution up and down 10 times with a 1 ml pipette until no tissue pieces are visible. After mechanical dissociation add to the mixture 400 µl ovomucoid-EBSS (10% v/v) a papain inhibitor to arrest further chemical dissociation.

For retinae from young mice (P14 and P25) digestion times were adapted from 20 to 10 min to compensate for a faster dissociation. For brain cortical cells, above described digestion was preceded by vibratome slicing of freshly obtained mouse brains.

Neuro-2a cells were trypsinized (0.05% Trypsin-EDTA, Thermo Fisher Scientific) and washed once with cold PBS. The relevant details of the cell lines used have been included in the Key resource table. The Neuro-2a (Mouse neuroblastoma) cells were obtained as frozen vials supplied and quality controlled by DSMZ, Germany (ACC-148; RRID: CVCL_0470). Cells were purchased in the year 2010, but were not long term cultured since then. Instead they had been stored in liquid nitrogen for the predominant amount of time and were only thawed days before the sorting experiment. In general cell culture facilities are regularly checked for bacterial infections, including mycoplasma infections, and there is no evidence suggesting an infection of the cells. Notably, cells were not recultured after FACS characterisation. In all cases the samples were filtered into Falcon round bottom polystyrene FACS tubes (Corning Inc, USA) using a 40 µm mesh cell strainer (FALCON, Corning Inc, USA) prior to FACS analysis.

For the calculation of the volume specific scattering, the side scattering area was normalized by volume of nuclei by taking the forward scattering area as a measure for size. Volume-specific scattering thus refers to the light scattering normalized by the amount of material, used to compare the light scattering by a material of given volume/mass but different size distribution.

### Mie models of nuclei

The scattering intensity calculations for the multi-chromocenter-nuclei depicted in Figure 1H, were performed using Mie scattering models of spheres in a refractive index contrast of 2% (Kreysing et al., 2010), the reported contrast of refraction between heterochromatin and euchromatin. Mie calculations were implemented via a MATLAB script (Mätzler, 2002) that can be downloaded at the following link - https://omlc.org/software/mie/. The relevant parameters used were m_euchromatin/medium = 1.02, m_heterochromatin = 1.04 (Kreysing et al., 2010) which are refractive index of the euchromatin/medium and heterochromatin/particles respectively. The wavelength used was 500 nm and volume fraction vf = 0.3351. The diameter of particles used were in the range 0.9–4 µm. Relative scattering efficiencies for packed scatterers represented in Figure 3—figure supplement 2 (E) were calculated based on dependent scattering models (Twersky, 1978).

### Micro projection setup

Ex-vivo retinal transmission measurements were carried out using a dedicated custom built, automated optical setup. This micro-projection setup (Figure 3—figure supplement 1 (A)) consisted of two distinct optical paths, one containing projection optics (functioned akin to the optics of the eye) that relayed images displayed by the projector LCD on to the image plane of the projection objective lens, and a second that recorded the retina transmitted images. The light source used (ML505L3, Thorlabs) had a spectrum close to that of the sensitivity of the rods ~ 510 nm. The objective lens (NA = 0.45, NPL Fluotar, Leitz, Germany) was chosen to closely match the f# number of the mouse eye (f#~1; Geng et al., 2011), with an added option to narrow the incident angular spectrum for absolute transmission measurements. The projected image on the retina was then collected via an imaging/efflux objective (Olympus U PlanApo 20x 0.75/inf corr) and recorded on an Andor Zyla-5.5 sCMOS camera.

### Calculation of MTF

To quantify Modulation Transfer Functions (MTF) spatially extended sinusoidal stripe patterns of different spatial frequency were micro-projected using a custom optical setup (Figure 3—figure supplement 1A) and transmitted images were recorded. The MTF was calculated as the ratio of the contrast in the transmitted image and the projected image. With a customized digital projector setup, the implementation of the sinusoidal stripe projection became a straightforward analysis of the optical property for wide retinal regions (~625 µm x ~ 750 µm) (Figures. Figure 3—figure supplement 1A,C), Figure 3—figure supplement 2A-D). The projection of spatially extended images that display many periods is however also key to capture image veil, since scattering at large angles may reduce contrast not locally (from one peak into the neighboring minimum) but across multiple stripe periods of the test image. In industries MTF is predominantly used to assess various optical systems such as lens, cameras, displays etc. (Williams and Becklund, 1989). An advantage of the MTF approach over any spatial domain approach (i.e. PSF analysis) is that overall performance of a system with optical components in series can be conveniently described as a product of the MTFs of the individual components (Boreman, 2001). In particular, MTF describes the frequency domain performance of an optical system as a ratio of the contrasts in the output image to the input object as given below,

$$Contrast=\frac{I_{max}-I_{min}}{I_{max}+I_{min}}-\frac{I_{0,max}-I_{0,min}}{I_{0,max}+I_{0,min}};MTF\left(\xi \right)=\frac{{Contrast}_{image}(\xi )}{{Contrast}_{object}(\xi )}$$

where, I is the image intensity and ξ is the spatial frequency (number of stripes per unit distance).

Practically, the raw images of the stripe patterns were first flat field corrected using Fiji (Schindelin et al., 2012) to ensure no global changes in contrasts affected further calculations. Each image was then processed using built in functions in MATLAB by taking an average along the direction orthogonal to the contrast modulation. The resulting one-dimensional sinusoidal intensity pattern was fit to a sine wave to extract $I_{max}$ and $I_{min}$. (Figure 3—figure supplement 2A-D. Subsequently, the MTF was calculated according to the above formula. The MTF of the retina was then obtained by normalizing the measured MTF against the MTF of the optical setup alone. The differential readout of the transmitted image through the inversion arrested TG-LBR retina allows an explicit understanding of the optical impact of the inner retina and the outer nuclear layer architecture in relation to other ocular constituents, such as the lens and the reported optical properties of mouse eye in in vivo studies (Geng et al., 2011; de la Cera et al., 2006; van Oterendorp et al., 2011). As for the photoreceptors outer segments, their impact is minimal as they act as waveguides as described in previous ex vivo studies (Ohzu et al., 1972).Such an effect is also verified by our simulations.

### Calculation of Strehl ratio

Strehl ratio (SR) is a commonly used single number estimate of the optical performance of a system that can also be used to evaluate the optical performance of ocular components (Marsack et al., 2004; Thibos et al., 2003). The SR in the spatial domain is formally defined to be the ratio of the peak intensities of a PSF to that of a diffraction limited PSF (Strehl, 1895). In terms of the frequency domain analysis, one can more accurately calculate the SR by taking the volume under the Optical Transfer Function, albeit for systems with negligible phase transfer properties (as planar tissues), the volume under the MTF suffices to calculate the SR. This way the SR was calculated for each biologically independent sample by taking area under the frequency weighted MTF curve along the spatial frequency (Figure 3—figure supplement 2F).

### PSF measurements

The point spread function (PSF) measurements were carried out using a 40 µm pinhole (P40H, Thorlabs) acting as a point light source, such that the demagnified point projected on the retina was of the size about 3 µm. Raw images were corrected for background by subtraction of a dark frame in FIJI. Resulting images were normalized with respect to the integral intensity in the field of view (~80 µm by ~ 80 µm), and the central region with an ROI of 40 µm by 40 µm was cropped, averaged and displayed in false color.

### Diffuse transmission measurements

The measurements were carried out with the micro projection setup above such that a point source was projected through an effective NA of 0.05 on to the retina with a final size of ~30 µm diameter. The transmitted light was collected using an Olympus UPlanSApo 40x 1.25 NA silicone immersion objective lens and recorded on the camera. The fractional transmission of the samples was then calculated, after subtraction of a dark frame reference, based on the integrated intensity in the entire field of view compared to the intensity without the sample in place.

### Hiding power

The angular-weighted integrated scattering intensity is also known as hiding power. Specifically, hiding power is represented as the product of the efficiency of scattering (Q_sca_) and the directional weightage component, otherwise known as the anisotropy factor (g) (Johnsen, 2012). The theoretical calculations based on Mie models presented in Figures(1H, 2 F-G) were done using a MATLAB script reported by Mätzler (2002) that can be downloaded at the following link - https://omlc.org/software/mie/. The relevant parameters used were m_euchromatin/medium = 1.02, m_heterochromatin = 1.04 (Kreysing et al., 2010) which are refractive index of the euchromatin/medium and heterochromatin/particles respectively. The wavelength used was 500 nm and volume fraction vf = 0.3351. The diameter of particles used were in the range 0.92–4 µm.

### Optical reconstitution

Equal amounts (by weight) of silica beads of diameter 2 µm (MSS002) and 4.5 µm (MSS004a, lot obtained from Whitehouse Scientific) of RI 1.48 were dispersed in separate cuvettes containing glycerol-water mixture (RI = 1.43), the larger beads closely resembled the size of heterochromatin mass after chromocenter fusion, and the smaller beads corresponded to a ~ 12 chromocenter case nucleus (at a conserved total volume/mass). An edge was imaged through the two dispersions using a commercial mobile phone camera with a LED white lights acting as a light source.

### Tissue preparation for optical characterization

Animals were sacrificed by cervical dislocation, and one eyeball immediately removed and opened in fresh environmentally oxygenated PBS. Next, the anterior of the eye, including the cornea and the lens, was fully removed. The retina was gently detached from the choroid, the optic nerve clipped and pulled out from the posterior cup. The retinal cup was placed on a 22 × 60 mm coverslip. Special attention was given to remove any residual vitreous humour sticking to the retina. While the retina remained floated in PBS radial incisions were made and the retinas were flattened on the coverslip by aspiring tiny amounts of the PBS. An appropriately flattened retina was mounted under a smaller coverslip in PBS. A 255 µm spacer was placed between the two coverslips under a stereomicroscope to prevent squeezing of the retina. Preparation were typically achieved in 2 min, and no retina was considered for measurement with a preparation time of more than 5 min. Optical measurements were done in an automated fashion with results in adult WT mice comparable to double pass experiments in vivo (Artal et al., 1998).

### Behavioral assessment - Optomotor response

Visual behavioral response was assessed using a fully automated, monitor based optomotor drum setup obtained from Striatech (Striatech GmbH, Tübingen, Germany) and the experiments were conducted at the CRTD, TU Dresden, Germany. The optomotor setup was a closed box with four digital displays to simulate a rotating cylinder of stripe patterns. An opening above allowed the view of the animal via a camera. An independent computer-controlled software was used to track the mice on the platform. The presentation of the pattern and scoring of the movement tracking performance was done through a proprietary software program. Software details can be found in Benkner et al. (2013).

Age (5–6 months old) and gender matched mice from Wild type and TG-LBR (transgenic) mice were used for comparison of the behavior. The tests were performed under three different lighting conditions of 70 Lux (Photopic), 20 mLux and 2 mLux (Scotopic). Based on parameters reported previously in similar behavioral experiments (Umino et al., 2008), bar stripe patterns were presented at a speed of 15 deg/s for various spatial frequencies ranging from 0.01 to 0.44 cycles/deg in photopic condition. For the scotopic conditions, the stimulus was maintained at a constant temporal frequency of 0.73 Hz and spatial frequency in the range 0.01–0.3 cycles/deg. The temporal frequency here refers to the combination of spatial frequency (cyc/deg) and speed of movement in (deg/s), which gives an effective temporal frequency, namely the change of contrast at a given point on the screen, which was maintained constant at a particular temporal frequency (0.73 cyc/s or Hz). The contrast of the object displayed on the screen was in the range 100–2%. For determining the threshold contrast, display contrast was reduced in steps of 5% up to absolute contrasts of 10% and steps of 2% below 10% contrast. The sizes of stripes tested were (6, 8, 11, 22, 33, 44, 55, 66, 88, 95, 100, 106 cycles/360^o^). Each stimulus was presented for a total of 30–35 s in sets of 5 s each with a gap of 5 s between each presentation. The direction of rotation of the stripes was altered between left and right for each subsequent trial and chosen at random for trial-1. Once the threshold contrast was experimentally determined, for statistical analysis purpose, response values for contrasts above the threshold were designated to be ‘yes’ response and values below the threshold as ‘no’ response.

### Nocturnal adaptation of behavioral testing setup

The ambient lighting of the test chamber for photopic condition was measured using a Lux meter (Testo 540). To reduce the lighting to scotopic levels appropriate ND filter sheets (ND 3.6, ND 4.8) were placed on the monitors. The ND filters were assembled by combining ND-1.2 filter sheets (e-color+ #299, Rosco Laboratories Inc). A custom made infrared light source was also installed to monitor and enable tracking of the animals on the platform under scotopic conditions. The Rho-/- mice were used as a control to ensure that the responses of the mice in the scotopic display test conditions purely relied on the rod visual pathway. 

### Modelling and simulation of light propagation

#### 2 -photon mapping of ONL model

Wild-type C57BL/6J mice were sacrificed by cervical dislocation. Immediately, eyes were enucleated and then cut in half around the equator, discarding all components of the eye but the posterior eye-cup. Retina was peeled off from the eye-cup. The retinal isolation was performed in paraformaldehyde (PFA) 4% in phosphate-buffered saline (PBS) solution and then left suspended to complete fixation for 20 min. The sample was then transferred to a PBS solution at 4°C after fixation. The fixed sample was deposited inside a TEFLON container and embedded in low melting agarose. The agarose embedded sample was sectioned adapting the method described previously (Clérin et al., 2014). The resulting retinal cross sections were stained with Hoechst 33342 and then wet mounted in a 50% glycerol/PBS solution using a No. 1 cover slip (Corning Inc, USA). Imaging was performed with confocal microscope (LSM 780, Zeiss Germany) in two-photon mode, equipped with a tunable pulsed infrared laser (Chameleon Vision II, Coherent, US) (excitation wavelength 730 nm, Objective: Zeiss LCI Plan-Neofluar 63x/1.3). The acquired intensity image was of size 190 × 190 × 82 µm with pixel-sizes of 83 × 83 × 250 nm in lateral dimensions and in depth.

### Image processing and segmentation of ONL model

To create a realistic refractive index map of packed nuclei within the ONL, the intensity image was first segmented into nuclei regions. To that end, a random forest classifier was trained via Fiji (Arganda-Carreras et al., 2017; Schindelin et al., 2012) to densely classify each pixel into background or foreground (nuclei). A watershed segmentation (van der Walt et al., 2014) was then applied on the probability map with manually generated seed points, resulting in 1758 individual nuclei instances. The refractive indices for these phases have been carefully estimated previously in single cell studies (Błaszczak et al., 2014; Kreysing et al., 2010; Schürmann et al., 2017; Solovei et al., 2009). Finally, the refractive index distribution inside each nuclei region was generated according to the two different models:

Inverted: Consisting of two refractive phases with n1 = 1.357 and n2 = 1.382, corresponding to euchromatin and heterochromatin, respectively. Each nuclei mask was split into shell and core regions of equal volume (via morphological shrinking operations on each mask), which were then assigned the respective refractive indices (n1 for shell, n2 for core).

Chromocenter: Here, 8–12 chromocenters were randomly picked within the nuclei mask and assigned points close to either the nuclei border or those chromocenters to the high refractive index phase (n2) until its joint volume reached half the full nuclei volume. The other points were then assigned the less dense refractive index n1.

The resulting refractive index distribution was then blurred in both cases with a small gaussian (sigma = 2 px) to create a smooth distribution. For both models it was furthermore ensured that both refractive phases occupied the same total volume.

### Light propagation simulations and scattering

Light propagation through both ONL models was simulated with GPU-accelerated scalar beam propagation method (Weigert et al., 2018). A computational simulation grid of size (1024,1024,645) with pixel-size 83 nm was used and the propagation of a plane wave (wavelength 500 nm) through the different ONL refractive index distributions was simulated. The surrounding was assumed to have a refractive index of n_0_ = 1.33. The integrated side scattering cross sections were calculated from the angular spectrum as per previous reports (Weigert et al., 2018).

### Relative contributions to MTFs from ONL & outer segments

In order to assess relative contribution of ONL and outer segments to the MTF of the retina dedicated simulations were carried out. These compared the scattering from chromocenters in an ONL (1 or 8 per nucleus), with outer segments that were simulated as cylinders that were 1.6 µm in diameter and 25 µm in length. The refractive index of the core of the outer segments was assumed to be 1.42 (Sidman, 1957). For the recorded simulations the scattering anisotropy factor and efficiency were extracted and converted into a frequency domain MTF data using appropriate theoretical models (Henyey and Greenstein, 1941; Wells, 1969). Results show that outer segments only have a negligible impact on the overall MTFs (Figure 3—figure supplement 2G), in agreement with previous experimental findings (Enoch, 1963) and models of the outer segment (Vohnsen, 2007; Vohnsen, 2014) acting as waveguides.

### Measurements of ocular parameters

Freshly excised mouse eye balls were imaged under a Olympus stereo microscope SZX16 equipped with a Q-imaging camera. The lens was also imaged under dark field conditions to better visualize the lens periphery. From the recorded images, the ocular parameters - lengths of the eye along two orthogonal axes were measured manually using FIJI. For the lens, mean feret diameter from the contour of the lens periphery was measured as an average estimate of the size of the lens.

### Data and materials availability

Data and specifications of simulations supporting the findings of this study are available via https://dx.doi.org/10.17617/3.3a. The biobeam software is available publicly from: https://maweigert.github.io/biobeam.

## Data Availability

Data and specifications of simulations supporting the findings of this study are available via: https://dx.doi.org/10.17617/3.3a. The biobeam software is available publicly from: https://maweigert.github.io/biobeam. The following dataset was generated: KaushikaramSubramanianMartinWeigertOliverBorschHeikePetzoldAlfonsoGarcia-UlloaEugeneW MyersMariusAderIrinaSoloveiMoritzKreysing2020Rod nuclear architecture determines contrast transmission of the retina and behavioral sensitivity in miceEdmond10.17617/3.3aPMC697435331825309

## Additional files

Transparent reporting form

## References

[bib1] Akimoto M, Cheng H, Zhu D, Brzezinski JA, Khanna R, Filippova E, Oh EC, Jing Y, Linares JL, Brooks M, Zareparsi S, Mears AJ, Hero A, Glaser T, Swaroop A (2006). Targeting of GFP to newborn rods by nrl promoter and temporal expression profiling of flow-sorted photoreceptors. PNAS.

[bib2] Alam NM, Altimus CM, Douglas RM, Hattar S, Prusky GT (2015). Photoreceptor regulation of spatial visual behavior. Investigative Opthalmology & Visual Science.

[bib3] Arganda-Carreras I, Kaynig V, Rueden C, Eliceiri KW, Schindelin J, Cardona A, Sebastian Seung H (2017). Trainable weka segmentation: a machine learning tool for microscopy pixel classification. Bioinformatics.

[bib4] Artal P, Herreros de Tejada P, Muñoz Tedó C, Green DG (1998). Retinal image quality in the rodent eye. Visual Neuroscience.

[bib5] Artal P, Chen L, Fernández EJ, Singer B, Manzanera S, Williams DR (2004). Neural compensation for the eye’s optical aberrations. Journal of Vision.

[bib6] Banks MS, Sprague WW, Schmoll J, Parnell JA, Love GD (2015). Why do animal eyes have pupils of different shapes?. Science Advances.

[bib7] Barlow HB (1956). Retinal noise and absolute threshold. Journal of the Optical Society of America.

[bib8] Becker JS, McCarthy RL, Sidoli S, Donahue G, Kaeding KE, He Z, Lin S, Garcia BA, Zaret KS (2017). Genomic and proteomic resolution of heterochromatin and its restriction of alternate fate genes. Molecular Cell.

[bib9] Benkner B, Mutter M, Ecke G, Münch TA (2013). Characterizing visual performance in mice: an objective and automated system based on the optokinetic reflex. Behavioral Neuroscience.

[bib10] Błaszczak Z, Kreysing M, Guck J (2014). Direct observation of light focusing by single photoreceptor cell nuclei. Optics Express.

[bib11] Boreman GD (2001). Modulation Transfer Function in Optical and Electro-Optical Systems.

[bib12] Boudard DL, Acar N, Bretillon L, Hicks D (2011). Retinas of the diurnal rodent Arvicanthis ansorgei are highly resistant to experimentally induced stress and degeneration. Investigative Opthalmology & Visual Science.

[bib13] Chang B, Hawes NL, Hurd RE, Davisson MT, Nusinowitz S, Heckenlively JR (2002). Retinal degeneration mutants in the mouse. Vision Research.

[bib14] Clérin E, Yang Y, Forster V, Fontaine V, Sahel J-A, Léveillard T (2014). Vibratome sectioning mouse retina to prepare photoreceptor cultures. Journal of Visualized Experiments.

[bib15] Cronin TW, Johnsen S, Marshall NJ, Warrant EJ (2014). Visual Ecology.

[bib16] de la Cera EG, Rodríguez G, Llorente L, Schaeffel F, Marcos S (2006). Optical aberrations in the mouse eye. Vision Research.

[bib17] de Vries HL (1943). The quantum character of light and its bearing upon threshold of vision, the differential sensitivity and visual acuity of the eye. Physica.

[bib18] Dowling JE (1987). The Retina.

[bib19] Drezek R, Guillaud M, Collier T, Boiko I, Malpica A, Macaulay C, Follen M, Richards-Kortum R (2003). Light scattering from cervical cells throughout neoplastic progression: influence of nuclear morphology, DNA content, and chromatin texture. Journal of Biomedical Optics.

[bib20] Dyer MA (2016). Lessons from retinoblastoma: implications for Cancer, development, evolution, and regenerative medicine. Trends in Molecular Medicine.

[bib21] Eberhart A, Kimura H, Leonhardt H, Joffe B, Solovei I (2012). Reliable detection of epigenetic histone marks and nuclear proteins in tissue cryosections. Chromosome Research.

[bib22] Eberhart A, Feodorova Y, Song C, Wanner G, Kiseleva E, Furukawa T, Kimura H, Schotta G, Leonhardt H, Joffe B, Solovei I (2013). Epigenetics of eu- and heterochromatin in inverted and conventional nuclei from mouse retina. Chromosome Research.

[bib23] Enoch JM (1961). Nature of the transmission of energy in the retinal receptors. Journal of the Optical Society of America.

[bib24] Enoch JM (1963). Optical properties of the retinal receptors*†. Journal of the Optical Society of America.

[bib25] Falk M, Feodorova Y, Naumova N, Imakaev M, Lajoie BR, Leonhardt H, Joffe B, Dekker J, Fudenberg G, Solovei I, Mirny LA (2019). Heterochromatin drives compartmentalization of inverted and conventional nuclei. Nature.

[bib26] Feodorova Y, Koch M, Bultman S, Michalakis S, Solovei I (2015). Quick and reliable method for retina dissociation and separation of rod photoreceptor perikarya from adult mice. MethodsX.

[bib27] Field GD, Rieke F (2002). Nonlinear signal transfer from mouse rods to bipolar cells and implications for visual sensitivity. Neuron.

[bib28] Flevaris AV, Murray SO (2015). Attention determines contextual enhancement versus suppression in human primary visual cortex. Journal of Neuroscience.

[bib29] Frohns A, Frohns F, Naumann SC, Layer PG, Löbrich M (2014). Inefficient double-strand break repair in murine rod photoreceptors with inverted heterochromatin organization. Current Biology.

[bib30] Geng Y, Schery LA, Sharma R, Dubra A, Ahmad K, Libby RT, Williams DR (2011). Optical properties of the mouse eye. Biomedical Optics Express.

[bib31] Henyey LC, Greenstein JL (1941). Diffuse radiation in the galaxy. The Astrophysical Journal.

[bib32] Hess RF, Dakin SC, Field DJ (1998). The role of "contrast enhancement" in the detection and appearance of visual contours. Vision Research.

[bib33] Hiler D, Chen X, Hazen J, Kupriyanov S, Carroll PA, Qu C, Xu B, Johnson D, Griffiths L, Frase S, Rodriguez AR, Martin G, Zhang J, Jeon J, Fan Y, Finkelstein D, Eisenman RN, Baldwin K, Dyer MA (2015). Quantification of retinogenesis in 3D cultures reveals epigenetic memory and higher efficiency in iPSCs derived from rod photoreceptors. Cell Stem Cell.

[bib34] Hughes AE, Enright JM, Myers CA, Shen SQ, Corbo JC (2017). Cell Type-Specific epigenomic analysis reveals a uniquely closed chromatin architecture in mouse rod photoreceptors. Scientific Reports.

[bib35] Humphries MM, Rancourt D, Farrar GJ, Kenna P, Hazel M, Bush RA, Sieving PA, Sheils DM, McNally N, Creighton P, Erven A, Boros A, Gulya K, Capecchi MR, Humphries P (1997). Retinopathy induced in mice by targeted disruption of the rhodopsin gene. Nature Genetics.

[bib36] Imai R, Nozaki T, Tani T, Kaizu K, Hibino K, Ide S, Tamura S, Takahashi K, Shribak M, Maeshima K (2017). Density imaging of heterochromatin in live cells using orientation-independent-DIC microscopy. Molecular Biology of the Cell.

[bib37] Ivanova A, Signore M, Caro N, Greene ND, Copp AJ, Martinez-Barbera JP (2005). In vivo genetic ablation by Cre-mediated expression of diphtheria toxin fragment A. Genesis.

[bib38] Jaissle GB, May CA, Reinhard J, Kohler K, Fauser S, Lütjen-Drecoll E, Zrenner E, Seeliger MW (2001). Evaluation of the rhodopsin knockout mouse as a model of pure cone function. Investigative Ophthalmology & Visual Science.

[bib39] Johnsen S (2012). The Optics of Life.

[bib40] Kirby TJ, Lammerding J (2018). Emerging views of the nucleus as a cellular mechanosensor. Nature Cell Biology.

[bib41] Kreysing M, Boyde L, Guck J, Chalut KJ (2010). Physical insight into light scattering by photoreceptor cell nuclei. Optics Letters.

[bib42] Kyba CCM, Mohar A, Posch T (2017). How bright is moonlight?. Astronomy & Geophysics.

[bib43] Marina OC, Sanders CK, Mourant JR (2012). Correlating light scattering with internal cellular structures. Biomedical Optics Express.

[bib44] Marsack JD, Thibos LN, Applegate RA (2004). Metrics of optical quality derived from wave aberrations predict visual performance. Journal of Vision.

[bib45] Mattar P, Stevanovic M, Nad I, Cayouette M (2018). Casz1 controls higher-order nuclear organization in rod photoreceptors. PNAS.

[bib46] Mätzler C (2002).

[bib47] Miroshnikova YA, Nava MM, Wickström SA (2017). Emerging roles of mechanical forces in chromatin regulation. Journal of Cell Science.

[bib48] Mourant JR, Canpolat M, Brocker C, Esponda-Ramos O, Johnson TM, Matanock A, Stetter K, Freyer JP (2000). Light scattering from cells: the contribution of the nucleus and the effects of proliferative status. Journal of Biomedical Optics.

[bib49] Nagelberg S, Zarzar LD, Nicolas N, Subramanian K, Kalow JA, Sresht V, Blankschtein D, Barbastathis G, Kreysing M, Swager TM, Kolle M (2017). Reconfigurable and responsive droplet-based compound micro-lenses. Nature Communications.

[bib50] Nelson P (2017). From Photon to Neuron.

[bib51] Němec P, Cveková P, Burda H, Benada O, Peichl L (2007). Visual systems and the role of vision in subterranean rodents: Diversity of retinal properties and visual system designs. Subterranean Rodents: News From Underground.

[bib52] Nilsson D-E (2009). The evolution of eyes and visually guided behaviour. Philosophical Transactions of the Royal Society B: Biological Sciences.

[bib53] O'Carroll DC, Warrant EJ (2017). Vision in dim light: highlights and challenges. Philosophical Transactions of the Royal Society B: Biological Sciences.

[bib54] Ohzu H, Enoch JM, O'Hair JC (1972). Optical modulation by the isolated retina and retinal receptors. Vision Research.

[bib55] Peichl L (2005). Diversity of mammalian photoreceptor properties: adaptations to habitat and lifestyle?. The Anatomical Record Part A: Discoveries in Molecular, Cellular, and Evolutionary Biology.

[bib56] Prusky GT, West PW, Douglas RM (2000). Behavioral assessment of visual acuity in mice and rats. Vision Research.

[bib57] Prusky GT, Alam NM, Beekman S, Douglas RM (2004). Rapid quantification of adult and developing mouse spatial vision using a virtual optomotor system. Investigative Opthalmology & Visual Science.

[bib58] Ronneberger O, Baddeley D, Scheipl F, Verveer PJ, Burkhardt H, Cremer C, Fahrmeir L, Cremer T, Joffe B (2008). Spatial quantitative analysis of fluorescently labeled nuclear structures: problems, methods, pitfalls. Chromosome Research.

[bib59] Rose A (1948). The sensitivity performance of the human eye on an absolute scale. Journal of the Optical Society of America.

[bib60] Schindelin J, Arganda-Carreras I, Frise E, Kaynig V, Longair M, Pietzsch T, Preibisch S, Rueden C, Saalfeld S, Schmid B, Tinevez J-Y, White DJ, Hartenstein V, Eliceiri K, Tomancak P, Cardona A (2012). Fiji: an open-source platform for biological-image analysis. Nature Methods.

[bib61] Schmucker C, Schaeffel F (2004). A paraxial schematic eye model for the growing C57BL/6 mouse. Vision Research.

[bib62] Schürmann M, Cojoc G, Girardo S, Ulbricht E, Guck J, Müller P (2017). Three-dimensional correlative single-cell imaging utilizing fluorescence and refractive index tomography. Journal of Biophotonics.

[bib63] Shevell SK, Holliday I, Whittle P (1992). Two separate neural mechanisms of brightness induction. Vision Research.

[bib64] Sidman RL (1957). The structure and concentration of solids in photoreceptor cells studied by refractometry and interference microscopy. The Journal of Biophysical and Biochemical Cytology.

[bib65] Solovei I, Grasser F, Lanctot C (2007). FISH on histological sections. Cold Spring Harbor Protocols.

[bib66] Solovei I, Kreysing M, Lanctôt C, Kösem S, Peichl L, Cremer T, Guck J, Joffe B (2009). Nuclear architecture of rod photoreceptor cells adapts to vision in mammalian evolution. Cell.

[bib67] Solovei I (2010). Fluorescence in situ Hybridization (FISH) on Tissue Cryosections. Fluorescence in Situ Hybridization.

[bib68] Solovei I, Wang AS, Thanisch K, Schmidt CS, Krebs S, Zwerger M, Cohen TV, Devys D, Foisner R, Peichl L, Herrmann H, Blum H, Engelkamp D, Stewart CL, Leonhardt H, Joffe B (2013). LBR and lamin A/C sequentially tether peripheral heterochromatin and inversely regulate differentiation. Cell.

[bib69] Stephens AD, Banigan EJ, Adam SA, Goldman RD, Marko JF (2017). Chromatin and lamin A determine two different mechanical response regimes of the cell nucleus. Molecular Biology of the Cell.

[bib70] Stephens AD, Banigan EJ, Marko JF (2019). Chromatin's physical properties shape the nucleus and its functions. Current Opinion in Cell Biology.

[bib71] Sterling P, Laughlin S (2015). Principles of Neural Design.

[bib72] Strehl K (1895). Aplanatische und fehlerhafte abbildung im fernrohr. Zeitschrift Für Instrumentenkunde.

[bib73] Thibos LN, Hong X, Bradley A, Applegate RA (2003). Metrics of Optical Quality of the Eye.

[bib74] Thibos LN, Hong X, Bradley A, Applegate RA (2004). Accuracy and precision of objective refraction from wavefront aberrations. Journal of Vision.

[bib75] Thomas KN, Robison BH, Johnsen S (2017). Two eyes for two purposes: *in situ* evidence for asymmetric vision in the cockeyed squids *Histioteuthis heteropsis* and *Stigmatoteuthis dofleini*. Philosophical Transactions of the Royal Society B: Biological Sciences.

[bib76] Tinsley JN, Molodtsov MI, Prevedel R, Wartmann D, Espigulé-Pons J, Lauwers M, Vaziri A (2016). Direct detection of a single photon by humans. Nature Communications.

[bib77] Tkatchenko TV, Shen Y, Tkatchenko AV (2010). Analysis of postnatal eye development in the mouse with High-Resolution small animal magnetic resonance imaging. Investigative Opthalmology & Visual Science.

[bib78] Twersky V (1978). Acoustic bulk parameters in distributions of pair‐correlated scatterers. The Journal of the Acoustical Society of America.

[bib79] Umino Y, Solessio E, Barlow RB (2008). Speed, spatial, and temporal tuning of rod and cone vision in mouse. Journal of Neuroscience.

[bib80] van der Walt S, Schönberger JL, Nunez-Iglesias J, Boulogne F, Warner JD, Yager N, Gouillart E, Yu T, scikit-image contributors (2014). scikit-image: image processing in Python. PeerJ.

[bib81] van Oterendorp C, Diaz-Santana L, Bull N, Biermann J, Jordan JF, Lagrèze WA, Martin KR (2011). Light scattering and wavefront aberrations in in vivo imaging of the rat eye: a comparison study. Investigative Opthalmology & Visual Science.

[bib82] Villegas EA, González C, Bourdoncle B, Bonnin T, Artal P (2002). Correlation between optical and psychophysical parameters as a function of defocus. Optometry and Vision Science.

[bib83] Vohnsen B (2007). Photoreceptor waveguides and effective retinal image quality. Journal of the Optical Society of America A.

[bib84] Vohnsen B (2014). Modeling photoreceptor mosaic imaging as backscattering of light from multilayered discs.

[bib85] Wang L, Hiler D, Xu B, AlDiri I, Chen X, Zhou X, Griffiths L, Valentine M, Shirinifard A, Sablauer A, Thiagarajan S, Barabas ME, Zhang J, Johnson D, Frase S, Dyer MA (2018). Retinal cell type DNA methylation and histone modifications predict reprogramming efficiency and retinogenesis in 3D organoid cultures. Cell Reports.

[bib86] Warrant EJ (1999). Seeing better at night: life style, eye design and the optimum strategy of spatial and temporal summation. Vision Research.

[bib87] Warrant EJ (2017). The remarkable visual capacities of nocturnal insects: vision at the limits with small eyes and tiny brains. Philosophical Transactions of the Royal Society B: Biological Sciences.

[bib88] Warrant EJ, Locket NA (2004). Vision in the deep sea. Biological Reviews.

[bib89] Warrant E, Nilsson D-E (2006). Invertebrate Vision.

[bib90] Weigert M, Subramanian K, Bundschuh ST, Myers EW, Kreysing M (2018). Biobeam-Multiplexed wave-optical simulations of light-sheet microscopy. PLOS Computational Biology.

[bib91] Wells WH (1969). Loss of resolution in water as a result of multiple Small-Angle scattering. Journal of the Optical Society of America.

[bib92] Werner JS, Chalupa LM (2004). The Visual Neurosciences.

[bib93] Williams CS, Becklund OA (1989). Introduction to the Optical Transfer Function.

[bib94] Williams RW, Moody SA (2004). *Developmental and Genetic Control of Cell Number in the Retina*. The Visual Neurosciences.

