# Supplementary material for: Rod nuclear architecture determines contrast transmission of the retina and behavioral sensitivity in mice
Source: eLife. 2019 Dec 11;8:e49542. doi: 10.7554/eLife.49542 (PMC6974353; doi:10.7554/eLife.49542)
Supplement: Transparent reporting form [file elife-49542-transrepform.docx]

***eLife’s* transparent reporting form**

We encourage authors to provide detailed information *within their submission* to facilitate the interpretation and replication of experiments. Authors can upload supporting documentation to indicate the use of appropriate reporting guidelines for health-related research (see [EQUATOR Network](http://www.equator-network.org/%20)), life science research (see the [BioSharing Information Resource](https://biosharing.org/)), or the [ARRIVE guidelines](http://www.plosbiology.org/article/info:doi/10.1371/journal.pbio.1000412) for reporting work involving animal research. Where applicable, authors should refer to any relevant reporting standards documents in this form.

If you have any questions, please consult our Journal Policies and/or contact us: [editorial@elifesciences.org](mailto:editorial@elifesciences.org).

**Sample-size estimation**

- You should state whether an appropriate sample size was computed when the study was being designed
- You should state the statistical method of sample size computation and any required assumptions
- If no explicit power analysis was used, you should describe how you decided what sample (replicate) size (number) to use

Please outline where this information can be found within the submission (e.g., sections or figure legends), or explain why this information doesn’t apply to your submission:

Sample size was determined to be adequate based on the magnitude and consistency of measurable differences between groups. Rationale for data exclusions if any are stated with appropriate reasoning in the relevant Supplementary methods. Experimental reproducibility is reflected in the reported standard deviations and corresponding error bars in the measure of the mean for plotted data.

**Replicates**

- You should report how often each experiment was performed
- You should include a definition of biological versus technical replication
- The data obtained should be provided and sufficient information should be provided to indicate the number of independent biological and/or technical replicates
- If you encountered any outliers, you should describe how these were handled
- Criteria for exclusion/inclusion of data should be clearly stated
- High-throughput sequence data should be uploaded before submission, with a private link for reviewers provided (these are available from both GEO and ArrayExpress)

Please outline where this information can be found within the submission (e.g., sections or figure legends), or explain why this information doesn’t apply to your submission:

All our corresponding figure legends and captions include information of the number of biologically independent samples.

**Statistical reporting**

- Statistical analysis methods should be described and justified
- Raw data should be presented in figures whenever informative to do so (typically when N per group is less than 10)
- For each experiment, you should identify the statistical tests used, exact values of N, definitions of center, methods of multiple test correction, and dispersion and precision measures (e.g., mean, median, SD, SEM, confidence intervals; and, for the major substantive results, a measure of effect size (e.g., Pearson's r, Cohen's d)
- Report exact p-values wherever possible alongside the summary statistics and 95% confidence intervals. These should be reported for all key questions and not only when the p-value is less than 0.05.

Please outline where this information can be found within the submission (e.g., sections or figure legends), or explain why this information doesn’t apply to your submission:

The corresponding figure legends and captions reports the statistical measures. Statistical analysis for the behavioural study was done using SPSS Statistics. All other statistical analyses were done using MATLAB. In all reported statistics, un- paired two tailed Student’s t tests were used except for the behavioral study where Chi-square automatic interaction detection (CHAID) was used for determining statistical significance. The details of sample size and error estimates are mentioned in the corresponding figures or figure legends. In all cases the error bar represents 95% Confidence Intervals unless otherwise stated. The statistical analysis for the behavioral studies were performed according to the ARRIVE guidelines. Significance was assumed with *p<0.05, **p<0.01, ***p<0.001. Exact p values are also reported whenever possible.

(For large datasets, or papers with a very large number of statistical tests, you may upload a single table file with tests, Ns, etc., with reference to sections in the manuscript.)

**Group allocation**

- Indicate how samples were allocated into experimental groups (in the case of clinical studies, please specify allocation to treatment method); if randomization was used, please also state if restricted randomization was applied
- Indicate if masking was used during group allocation, data collection and/or data analysis

Please outline where this information can be found within the submission (e.g., sections or figure legends), or explain why this information doesn’t apply to your submission:

This research did not involve clinical studies or treatment in live animals. Group allocation was not performed. Mice analyzed were non-randomized litter mates, sex-matched whenever possible. Investigators were not blinded to mouse genotypes during experiments. Data reported for mouse experiments are not subjective but rather based on quantitative flow cytometry, quantitative image analysis and fully automated behavioral testing.

**Additional data files (“source data”)**

- We encourage you to upload relevant additional data files, such as numerical data that are represented as a graph in a figure, or as a summary table
- Where provided, these should be in the most useful format, and they can be uploaded as “Source data” files linked to a main figure or table
- Include model definition files including the full list of parameters used
- Include code used for data analysis (e.g., R, MatLab)
- Avoid stating that data files are “available upon request”

Please indicate the figures or tables for which source data files have been provided:

Data and specifications of simulations supporting the findings of this study are available via <https://owncloud.mpi-cbg.de/index.php/s/SaCJjsMCfyOAaTb>. The biobeam software is available publicly from: <https://maweigert.github.io/biobeam>
